# Conceptual framework for tinnitus: a cognitive model in practice

**DOI:** 10.1038/s41598-023-48006-7

**Published:** 2024-03-26

**Authors:** Iman Ghodratitoostani, Zahra Vaziri, Milton Miranda Neto, Camila de Giacomo Carneiro Barros, Alexandre Cláudio Botazzo Delbem, Miguel Angelo Hyppolito, Hamid Jalilvand, Francisco Louzada, Joao Pereira Leite

**Affiliations:** 1 Neurocognitive Engineering Laboratory (NEL), Center for Engineering Applied to Health, Institute of Mathematics and Computer Science, University of Sao Paulo, Sao Carlos, Brazil; 2 Department of Neurosciences and Behavioral Sciences, Medical School of Ribeirão Preto, University of São Paulo, São Paulo, Brazil; 3 Institute of Mathematics and Computer Science, University of São Paulo, São Carlos, Brazil; 4 Department of Otorhinolaryngology, Ribeirão Preto Medical School, Universidade de São Paulo, Ribeirão Preto, Brazil; 5 Department of Ophthalmology, Otorhinolaryngology, Head and Neck Surgery, Ribeirão Preto Medical School, University of São Paulo, São Paulo, Brazil; 6 Department of Audiology, School of Rehabilitation, Shahid Beheshti University of Medical Sciences, Tehran, Iran; 7 Present Address: Adjunct Scholar, Tehran University of Medical Sciences, Tehran, Iran

**Keywords:** Predictive medicine, Cortex, Attention, Prefrontal cortex, Limbic system, Sensory processing

## Abstract

Tinnitus is a conscious attended awareness perception of sourceless sound. Widespread theoretical and evidence-based neurofunctional and psychological models have tried to explain tinnitus-related distress considering the influence of psychological and cognitive factors. However, tinnitus models seem to be less focused on causality, thereby easily misleading interpretations. Also, they may be incapable of individualization. This study proposes a Conceptual Cognitive Framework (CCF) providing insight into cognitive mechanisms involved in the predisposition, precipitation, and perpetuation of tinnitus and consequent cognitive-emotional disturbances. The current CCF for tinnitus relies on evaluative conditional learning and appraisal, generating negative valence (emotional value) and arousal (cognitive value) to annoyance, distress, and distorted perception. The suggested methodology is well-defined, reproducible, and accessible, which can help foster future high-quality clinical databases. Perceived tinnitus through the perpetual-learning process can always lead to annoyance, but only in the clinical stage directly cause annoyance. In the clinical stage, tinnitus perception can lead indirectly to distress only with experiencing annoyance either with (“${\underset{{\mathcal {C}}}{{{\varvec{Ind-1}}}}}$” = 1.87; 95% CI 1.18–2.72)[“1st indirect path in the Clinical stage model”: Tinnitus Loudness $\rightarrow$ Attention Bias $\rightarrow$ Cognitive-Emotional Value $\rightarrow$ Annoyance $\rightarrow$ Clinical Distress]or without (“${\underset{{\mathcal {C}}}{{{\varvec{Ind-2}}}}}$”= 2.03; 95% CI 1.02–3.32)[ “2nd indirect path in the Clinical stage model”: Tinnitus Loudness $\rightarrow$ Annoyance $\rightarrow$ Clinical Distress] the perpetual-learning process. Further real-life testing of the CCF is expected to express a meticulous, decision-supporting platform for cognitive rehabilitation and clinical interventions. Furthermore, the suggested methodology offers a reliable platform for CCF development in other cognitive impairments and supports the causal clinical data models. It may also enhance our knowledge of psychological disorders and complicated comorbidities by supporting the design of different rehabilitation interventions and comprehensive frameworks in line with the “preventive medicine” policy.

Tinnitus is a Continuous Attended Awareness Perception (CAAP) of sourceless sound. A recent systematic review discovered that around 10% of young adults, 14% of middle-aged adults, and 25% of older adults commonly experience auditory phantom perception^1^. Based on clinical observations, It currently remains unclear why only 17% of tinnitus perceivers are experiencing bothersome^2^. Several theoretical, cognitive, and behavioral models have sought to explain the influence of psychological factors in originating or mitigating tinnitus distress^3–10^. Corresponding clinical evidence for each model is shown in the supplementary documents “*Table of the Clinical evidence*”. The habituation model proposed by Hallam *et al.*^6^ suggests a psychological description for distressing tinnitus. These authors stated that the negative interpretation of the tinnitus sound and its associated elevated autonomic arousal levels causes dysfunctional cognitive processing, thereby disrupting habituation to the perception of the tinnitus sound.

Jastreboff^7^ posited the neurophysiological model that reports that classical conditioning could be the dominant mechanism behind the aversive emotional states of tinnitus. Zenner and Zalaman^10^ developed the Cognitive Desensitized Therapy that revealed a reduction in psychosomatic tinnitus burden and the daily observation time and an improvement in the quality of life^10^. Moreover, Zenner *et al.*^9^ postulated that tinnitus sensitization develops during the interpretation of perceiving sound as unpredictable, noxious, and fear-inducing results in the sense of deficiency in coping and helplessness^9,10^. McKenna *et al.*^8^ proposed the Cognitive-Behavioral model of tinnitus that the process of distress begins with intrusive, excessive negative thoughts about perceived tinnitus sound. These negative thoughts provoke arousal and emotional distress and drive factors such as selective attention, monitoring, and counterproductive safety behaviors, all of which exacerbate tinnitus distress. McKenna *et al.*^8^ hypothesized that cognitive misinterpretation of the tinnitus results in distress and physiological arousal, causing distorted perception from sensory input^8^.

Recently, Ghodratitoostani *et al.*^4^ postulated the Neurofunctional Tinnitus Model (NfTM) and emphasized that the CAAP of tinnitus is crucial for causing distress. NfTM characterized tinnitus patients into the following two stages:

- *Neutral stage*: perceiving tinnitus without distress reaction
- *Clinical distress stage*: experiencing distress reaction due to the corresponding negative valence when the tinnitus is perceived^3,4^.

Valence denotes emotional states ranging along a continuum from positive to negative feelings with a neutral Midpoint^11^. Tinnitus-related valence progressively shifts to negative through the Evaluative Conditional Learning (ECL) wherein repeated pairing of neutral tinnitus conditioned with similar or different negative unconditional stimuli promotes negative valence^3,12^.

Appraisal and ECL mechanisms excite tinnitus-related cognitive-emotional value and drive preferential attention allocation to the sound and prolonged tinnitus perception. On the other hand, negative appraisals such as “the noise makes my life unbearable”, “it will drive me crazy”, “it will overwhelm me”. Handscomb *et al.*^13^ intermittently strengthen the cognitive value of tinnitus. Contrarily, NfTM posits that the CAAP of tinnitus concurrent with presenting positively-valenced stimuli might change negative valence, resulting in less frequent tinnitus perception with a lower level of distress^3^. Cognitive functions proposed in NfTM can also be embodied in the emotion regulation process^14,15^ of tinnitus, which suggests that the tinnitus loudness misperception may link to the negative valence and selective attention^16^. NfTM also posits that perpetual evaluation of tinnitus valence and its comparison with the valence of sensory inputs in the same and different modalities occur in the prefrontal cortex^3,17^.

To our knowledge, causal relationships have been rarely explored in the literature, which can easily lead to misinterpretations of the findings. Therefore, introducing approaches to conceptualizing causal relationships and hypotheses is essential for reliable interpretations. A novel tinnitus theoretical-conceptual model enables the drawing of data models for testing causality relationships between independent variables and outcomes within retrospective studies. It can navigate research strategies in prospective studies on tinnitus cognitive rehabilitation^18^.

The current paper attempts to fill this void by proposing and validating a novel Conceptual Cognitive Framework (CCF) for tinnitus in light of the previous models. This model draws heavily from cognitive processes proposed by NfTM^3,16^), and the modal model of emotion Gross^19^. CCF illustrates cognitive processes and their interactions, contributing to the development and maintenance of annoyance-distress reactions based on the tinnitus stage (neutral vs. clinical). Thereafter, we provided support from literature for the components of the CCF. More importantly, multi-mediatory (causality) modeling approaches^20^ also demonstrate the tinnitus causality model.

## Proposed conceptual cognitive framework

### Fundamental ideas and postulations of conceptual cognitive framework

The following are the assumptions behind the proposed model:

- CCF aims to illustrate associations between cognitive processes causing annoyance-distress reactions in tinnitus.
- CCF primarily rests on Evaluative Conditioning and assumes that concurrent CAAP into Unconditional Stimulus (US), Conditional Stimulus (CS), and their contingencies is essential for attitude formation^3,12^.
- Either or both negative cognitive and emotional values can cause annoyance, though they can affect each other merely through annoyance. The annoyance affects both cognitive value and emotional value, distorting the perception of tinnitus^16^.
- In the neutral stage, the negative cognitive-emotional values could generate annoyance, but they are insufficient to trigger distress reactions. Accordingly, annoyance and distress are considered two different concepts.

**Figure 1:**
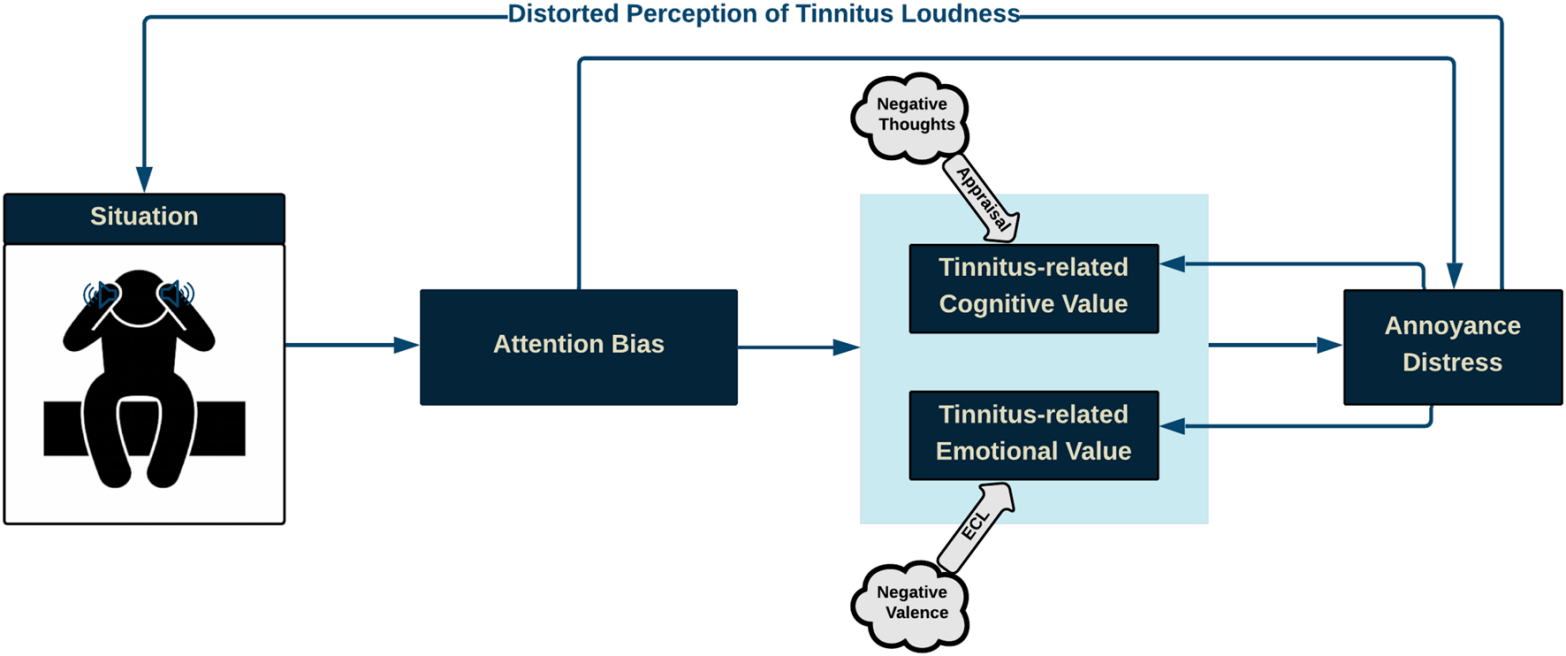
Conceptual cognitive framework of tinnitus; tinnitus CCF speculates that in the pre-sleep situation when tinnitus-related cues (emotionally-laden or relevant to individuals’ concerns) capture attention. Then, either directly or through tinnitus-related cognitive and emotional values, triggers an annoyance-distress mechanism leading to a distorted perception of tinnitus loudness, exacerbating the tinnitus experience. Likewise, tinnitus distress reinforces the negative cognitive-emotional value of tinnitus.

Hypothetically, CCF compartments include situation, attention bias, cognitive value (arousal), emotional value (valence), annoyance-distress reaction, and distorted perception. The proposed CCF aims to illustrate the interaction between cognitive processes that contributes to generating distress reactions. The current study concentrated on tinnitus experienced in silence and before sleep. CCF postulates that stimuli related to tinnitus preferentially capture attention, either directly or through corresponding cognitive and emotional values, triggering annoyance or distress reaction leading to distorted perception. In turn, distress feeds back to and influences the situation. Likewise, distress reaction fuels back corresponding cognitive and emotional values. Figure 1 illustrates the proposed CCF.

To provide proof of concept for the proposed CCF, we primarily present supporting studies from Tinnitus literature. We then explore Multi-Mediation models to examine proposed causal relationships between cognitive and emotional factors in the CCF.

## Compartments and cognitive processes

### Situation

Nighttime silence at the pre-sleep period could facilitate CAAP of internal [tinnitus sound, body sensation or thoughts] and external [environmental sounds, light, heat] stimuli. Asnis *et al.*^21^ documented that in the absence of environmental noises, the perception of tinnitus sound facilitates and interferes with the process of falling asleep or getting back to sleep^21,22^.

Using a mobile application, Probst *et al.*^23^ performed an investigation on Tinnitus patients’ daily life and revealed that the environmental sound level differs based on the day’s time. Most severe tinnitus loudness and distress were experienced at night and early morning hours (12 a.m. midnight to 8 a.m.) because of lower environmental sound levels, which could not attenuate tinnitus perception in these interims^23^.

### Attention bias

CAAP of internal and external stimuli shape individuals’ expectations and predictions from the pre-sleep situation. Thus, any novelty or changes in the features of the aforementioned stimuli can bias attention^24^. This finding was corroborated by Roberts *et al.*^25^ and Winkler *et al.*^26^, who suggested that discrepancy between an expectation and upcoming stimuli could bias attention”^24^. Furthermore, emotionally-laden or threat-related stimuli can also have priority over other stimuli, leading to attentional bias, similar to what cognitive theories of anxiety disorders had proposed^27^. According to this view, prioritized attention allocation to threat cues may lead to developing and maintaining anxiety^28^. The threat cues for patients with tinnitus could be related to tinnitus characteristics [CAAP of tinnitus sound or changes in the tinnitus loudness or pitch] which impair the process of falling asleep as the active goal of pre-sleep time.

Emotional Stroop Task (EST) is one of the most-used paradigms to experimentally assess attentional bias, in which a set of emotional words (relevant to the subjects’ clinical condition) and neutral words (irrelevant) shown in different colors are presented to the participants. The participants respond to the color of the words as quickly as possible by pressing the corresponding key on the keyboard while ignoring the words’ meaning. Prolonged responses to the color of emotionally-laden words compared with neutral words suggest biasing of attentional resources towards the emotionally-laden information^29^. A limited number of studies assessing attentional bias on the tinnitus population through the EST and the findings remain inconclusive and inconsistent. Andersson *et al.*^30^ reported a faster reaction time to tinnitus-related words, and other researchers could not find a Stroop effect in tinnitus patients’ reaction time^31,32^. However, inconsistent results in tinnitus studies emerge from confounding factors and possible biases effects on imperfect methodologies that were applied in the study design like in Andersson *et al.*^30^ study with a remarkable difference in the sample size of the tinnitus group ($n=104$) and healthy group ($n=21$). Moreover, the salience of the applied emotional words is inadequate to interfere with the task.

Therefore, utilizing more personalized tinnitus words to guarantee the high individualized importance of the patients’ stimuli may offer better results. Paradigms that examine auditory selective attention or dichotic listening tasks could be more proper to find differences on a behavioral level^32^.

### Emotional value

The Emotional value builds up through the ECL mechanism, which plays a vital role in liking and disliking stimuli^3^. Based on ECL, neutral stimuli (CS) can obtain positive or negative valence after frequently pairing with emotion-laden stimuli (US)^12^. Valence describes the emotional states varying along a continuum from positive to negative feelings with a neutral midpoint^11^. Based on CCF, the CAAP of both CS and US and their contingencies are essential at ECL formation. Moreover, evaluative conditioning is an accumulative procedure so that different valenced US stimuli can aggregate to CS valence over intermittent pairing^33^. Accordingly, ECL is resistant to extinction so that neither of CS/US only presence nor pairing CS with the different Unconditional Stimulus (US) would extinguish formerly shaped ECL memory^12^. Based on CCF, the negative valence of other US, through the ECL mechanism, fuels the negative tinnitus-related emotional value causing annoyance or distress reaction. The frequent co-occurrence of tinnitus sound and the negative US enhance negative tinnitus-related emotional value. Therefore, tinnitus perception alone could trigger distress reactions due to the shaped learning memory of the US’s valence^3^.

### Cognitive value

The cognitive value of perceiving internal and external stimuli develops through the appraisal process. When the meaning of an object or event is judged in a particular situation based on beliefs, desires, and intentions, the appraisal process engages^34^. However, only stimuli relevant to the individuals’ concern^35^ can trigger a cognitively aroused state followed by appraisal. Accordingly, attentional bias to tinnitus sound cues, as a concern-relevant stimulus, can provoke a cognitively-aroused state and resultant appraisals about tinnitus,“*If only the noise would go away*,” “*Why me? Why do I have to suffer this horrible noise?*”^36^. Negative thoughts through appraisal mechanisms fuel the negative tinnitus-related cognitive value leading to annoyance or distress reaction. Self-report questionnaires are widely employed to collect patients’ thoughts and beliefs about events, situations, or objects, depicting the role of CAAP in appraisals to examine conditions and their respective consequences. Tinnitus Cognitions Questionnaire^36^ assesses the content and the frequency of positive and negative thoughts associated with tinnitus. Wilson and Henry^36^ asked 200 tinnitus subjects to specify how frequently they experience thoughts in Tinnitus Cognitions Questionnaire. The highest endorsement rates were for the following negative statements: “*If only the noise would go away*,” “*Why me? Why do I have to suffer this horrible noise?*” “*I cannot enjoy what I am doing because of the noise,*” “*The noise will drive me crazy*”^36^. Studies also reported that negative thoughts were independent of positive thoughts suggesting that the absence of positive thoughts does not indicate negative thoughts. Furthermore, highly positive correlations were observed between negative Tinnitus Cognitions Questionnaire and depression, emotional distress, insomnia, and tinnitus handicap^36^. Other cognition aspects like catastrophizing, a tendency to exaggerate a problem’s negative aspects, are evaluated with the Tinnitus-Related Self-Statements Scale^37^ and Tinnitus Catastrophizing Scale^38^. Using Tinnitus Catastrophizing Scale, Cima *et al.*^38^ revealed that a catastrophic misinterpretation of tinnitus is strongly associated with enhanced fear and attention toward tinnitus and lower ratings of life quality. Similarly, Andersson *et al.*^39^ accomplished the experimental research on the effects of suppressing tinnitus-related thoughts (as an attention control strategy) to examine the immediate consequences of suppressing versus not suppressing thought (attending to tinnitus). Studies showed that Tinnitus-related thoughts were reduced by suppressive instructions while increased by attending to tinnitus. However, similar outcomes were not observed in tinnitus patients of the control group who neither suppressed nor attended to their tinnitus.

### Annoyance-distress reaction

In line with several cognitive-behavioral studies, CCF suggested that negative appraisal about tinnitus sound triggers annoyance-distress reactions. The cognitive-behavioral model of tinnitus^8^ projected that the process of distress begins with intrusive, overly negative thoughts about perceived tinnitus sounds. These negative thoughts provoke arousal and emotional distress and drive maintaining selective attention, monitoring, and counterproductive safety behaviors. Further studies corroborated that negative evaluations of tinnitus sound^40,41^ or catastrophic appraisals about it^42^ were associated with tinnitus distress severity. However, Heinecke *et al.*^43^ revealed that negative appraisal does not necessarily lead to physiological arousal, and he designed a crossover experiment that presented stress-inducing conditions to the group of tinnitus patients^43^. The results showed that the tinnitus group compared with the control group, reported higher emotional strain, while no differences were detected in stress physiological measures. The investigators attributed the mismatch between subjective self-reports and objective measurements to negative appraisal processes and catastrophizing thoughts in tinnitus patients^43^.

### Distorted perception

CCF proposed that valence and cognitive-arousal as two components of emotion can affect patients’ judgment about tinnitus pitch and loudness^16^. The following findings lend support to this hypothesis; Yoo and Lee^44^ studied the effect of modulating arousal and valence on time-perception in subjects with social anxiety, comparing the time duration of presented stimuli with the standard duration in training sessions. They showed that the duration of negative-stimuli against positive-stimuli was estimated longer with high arousal but shorter with low arousal levels. This finding suggests modification in the tendency and magnitude of valence and arousal modulates time-perception^44^. It can also be analogous with the tinnitus loudness perception versus annoyance. Durai *et al.*^45^ showed that emotional stimuli with weighted valence and arousal could affect tinnitus patients’ judgment in the rating of tinnitus annoyance and loudness, not in loudness match^45^. Likewise, psychological and Cognitive Behavioural Therapy (CBT) studies on tinnitus expressed significant improvement in tinnitus distress or tinnitus-related quality of life, while tinnitus loudness match did not change^46,47^.

### Hypotheses of conceptual cognitive framework

We hypothesized that in the Neutral stage, failure in ignorance, together with negative cognitive-emotional valuation, are essential for CAAP of phantom sound to generate annoyance but not sufficient to cause distress (Continuous-Learning-Path). Moreover, in the Clinical stage, we postulated that CAAP of tinnitus sound directly or through “Continuous-Learning-Path” enhances annoyance, ending with distress.

Next, we propose that in the Clinical stages, intermittent distress experience leads to a misperception about tinnitus loudness.

## Tinnitus causality model

Tinnitus Conceptual Cognitive Framework (CCF) attempts to illustrate causality (mediation) relationships. Hence, a methodology is required to evaluate causalities in theories and hypotheses. The tinnitus CCF supports data models for testing mediational relationships between independent variables and outcomes measured in retrospective studies. We demonstrated causality relationships through multi-mediatory (causality) modeling approaches^48^ for Neutral (learning process) and Clinical (maintenance) Tinnitus. We employed mediator modeling to put the concept of tinnitus-CCF into practice. Table 1 shows the items selected from each questionnaire to develop the tinnitus Mediator-Causality model.

**Table 1:** List of Questionnaires and corresponding questions and scores for model components.

Questionnaire	Item	Model component	Abbr
Tinnitus Handicap Inventory (THI)	Final score of questionnaire	Distress	THIR
Tinnitus Severity (TS)	$Q_2$. How strong or loud is your tinnitus at present?	Tinnitus loudness	Ts2
	$Q_3$. How uncomfortable is your tinnitus at present if everything around you is quiet?	Cognitive value	Ts3
	$Q_4$. How annoying is your tinnitus at present?	Annoyance	Ts4
	$Q_5$. How easy is it for you to ignore your tinnitus at present?	Attention Bias	Ts5
	$Q_6$. How unpleasant is your tinnitus at present?	Emotional value	Ts6
	Average of [$Q_3$ and $Q_6$]	Cognitive-Emotional Value	Ts36

Allgaier et al.^49^ used similar questions for daily monitoring tinnitus in the “TrackYourTinnitus” project.

**Figure 2:**
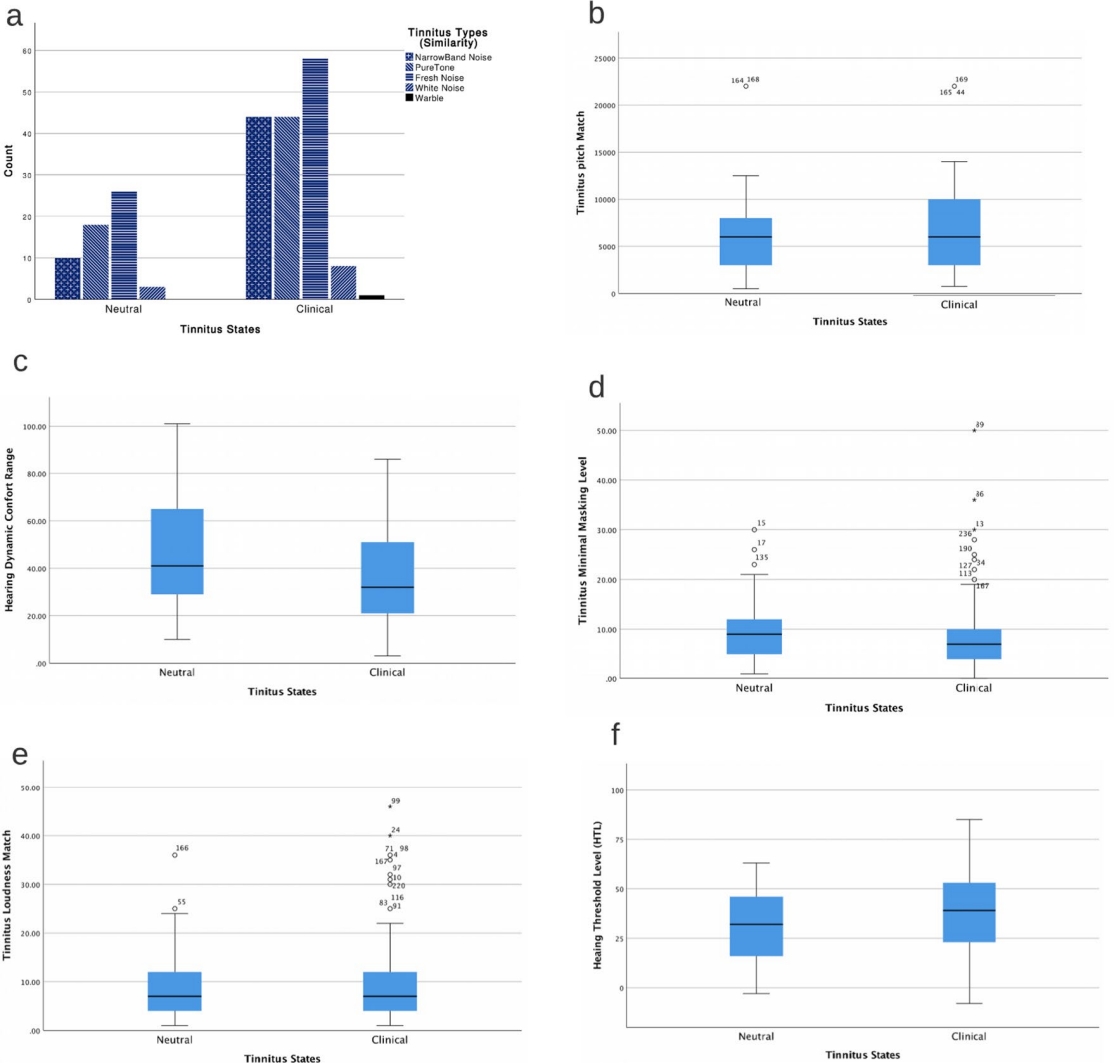
Descriptive analytics of psychoacoustic parameters were depicted in different segments (Neutral and Clinical) of the applied database; (**a**) Tinnitus types (similarity) include narrow band noise, pure-tone, fresh noise, white noise, and warble recognizing in different bars and textures. (**b**) Tinnitus pitch, (**c**) Hearing dynamic comfort range, which calculates from hearing threshold and loudness discomfort level, (**d**) Tinnitus minimal masking level, (**e**) Tinnitus loudness match, and (**f**) Hearing threshold level at the tinnitus pitch of the tinnitus type. For clinical procedures, review supplementary data^16^.

**Figure 3:**
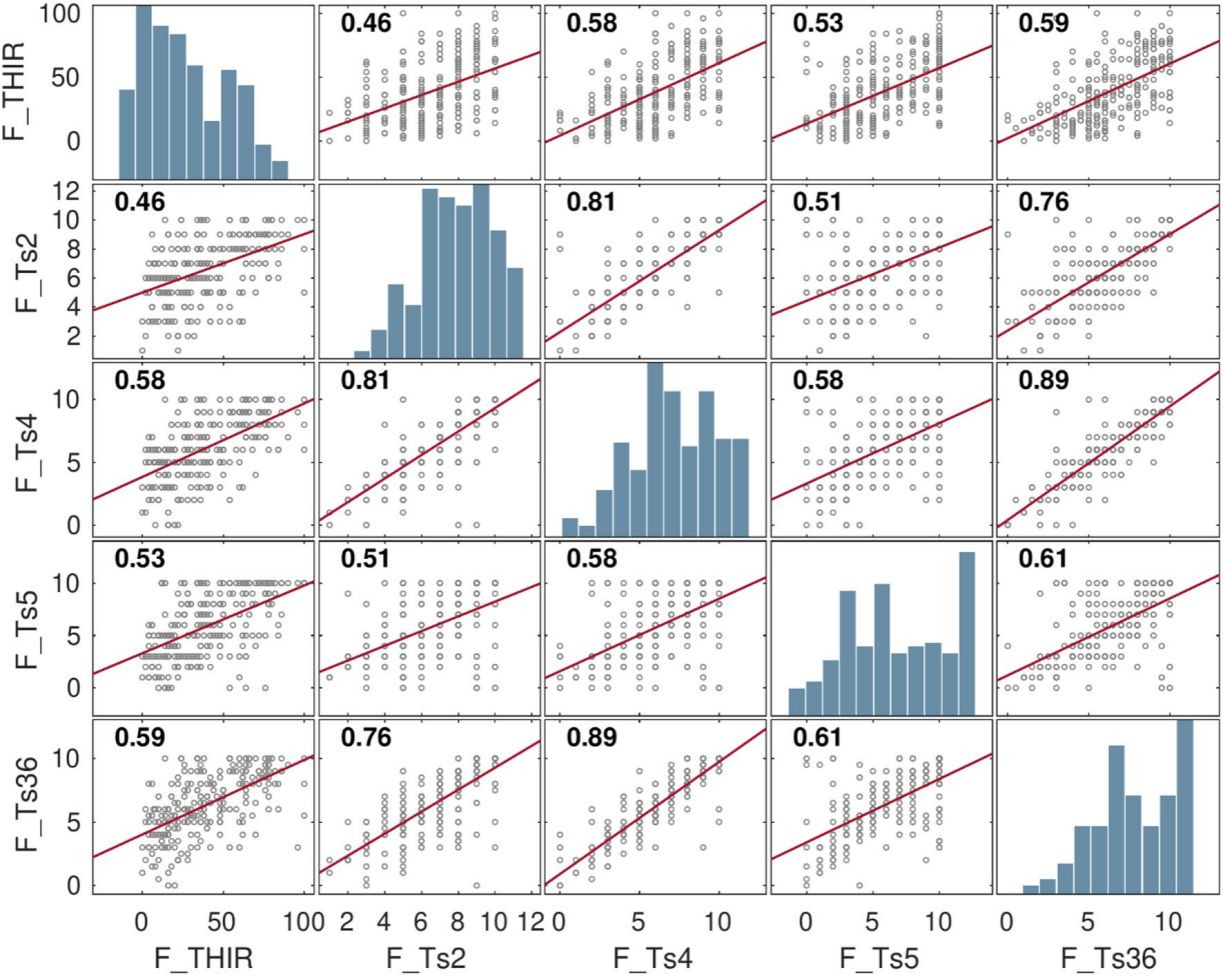
Correlation Matrix of variables used in the causal model of tinnitus to support evidence for CCF of tinnitus. Corresponding variables named Ts2: Tinnitus Loudness, Ts4: annoyance, Ts5: Attention Bias, Ts36: Cognitive-Emotional value, and THIR: Tinnitus Distress were already depicted in Table 1 Variables received “F” as representing suffix for Full Dataset.

**Figure 4:**
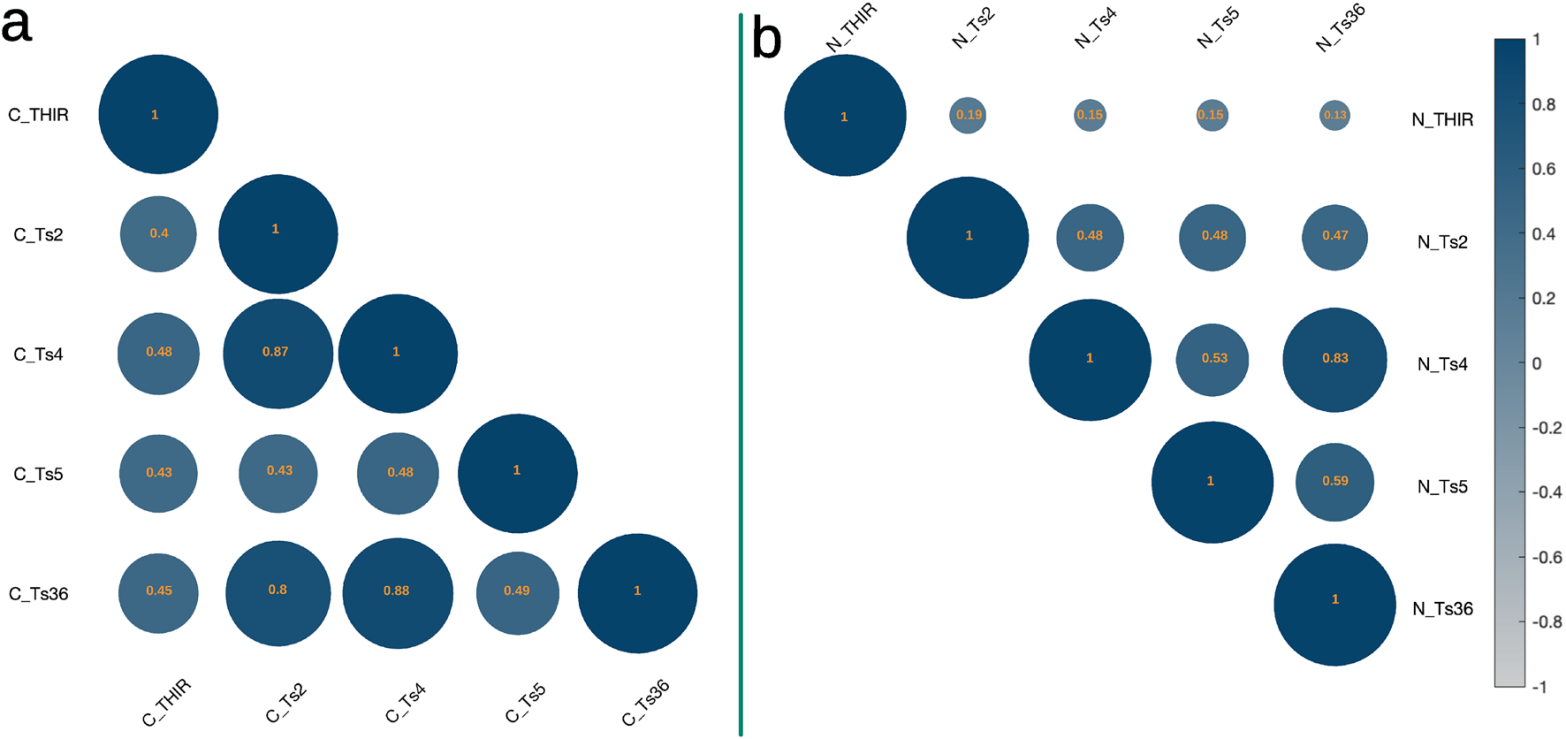
The correlation Matrix of variables used in the causal model of tinnitus supports CCF of tinnitus, Corresponding variables named Ts2: Tinnitus Loudness, Ts4: annoyance, Ts5: Attention Bias, Ts36: Cognitive-Emotional value, and THIR: Tinnitus Distress were already depicted in Table 1; (**a**) depicts the lower triangular matrix of the Clinical segment of the dataset, and corresponding variables received “C” as a suffix, and (**b**) indicates the upper triangular matrix of the Neutral segment of the dataset. Corresponding variables received “N” as a suffix. The circles’ color and size indicate direction and correlation coefficient value, shown in circles.

### Data Preprocessing

For the CCF assessment, data were collected from the participants of (1) an observational prospective cohort study and (2) a randomized crossover three-session double-blind study. The Ethics Committee approved both studies for Analysis of Research Projects, Specialized Center of Otorhinolaryngology and Speech Therapy, Hospital das Clínicas de Ribeirão Preto, University of São Paulo, Brazil (HCRP no 09813519.1.0000.5440; internationally registered with U1111-1236-5441, and HCRP no 55716616.1.1001.5440), All recruited patients signed written informed consent approved by HCRP and conducted under the standards specified in the 1964 Declaration of Helsinki. Literate patients with constant bilateral subjective tinnitus, normal hearing, or utmost moderate sensorineural hearing loss, normal color vision, and no history of psychoactive medication were included. Patients with pulsatile tinnitus, otosclerosis, Meniere’s disease, chronic headache, and other neurological disorders such as brain tumors and those treated for mental or central nervous system disorders were excluded. The data were anonymized to ensure blinding. Initially, those with missing values were omitted, resulting in 253 participants (123 female, 130 male) aged 27–72 years (54.43 ± 10.31 years) session-wised questionnaires from both studies^50^.

Before the sessions in both studies, participants filled up a Portuguese version of a battery of questionnaires that included (a) Tinnitus Handicap Inventory (THI) aims to identify, quantify, and evaluate the difficulties patients with tinnitus may experience^51^. (b) Tinnitus severity (TS) is a self-report 10-point Likert scale broadly used for tinnitus severity levels. Table 1 shows the items selected from each questionnaire for the development of the tinnitus Mediator-Causality model.

Initially, data sets were anonymized to ensure blinding and segmented based on different tinnitus severity stages. Scores of the THI questionnaire lower than 20 (THI-R < 20) were labeled as Neutral otherwise (THI-R $\ge$ 20) the Clinical.

Tinnitus evaluation on tinnitus was performed before each session Laterality, Similarity, and Pitch Matching Test (PMT). Jointly with Hearing Threshold Level (HTL), Loudness Match Test (LMT), Minimal Masking Level (MML), and Loudness Discomfort Level (LDL) for clinical procedures see supplementary data^16^. Comparative plots between two dataset segments are illustrated in Fig. 2.

**Figure 5:**
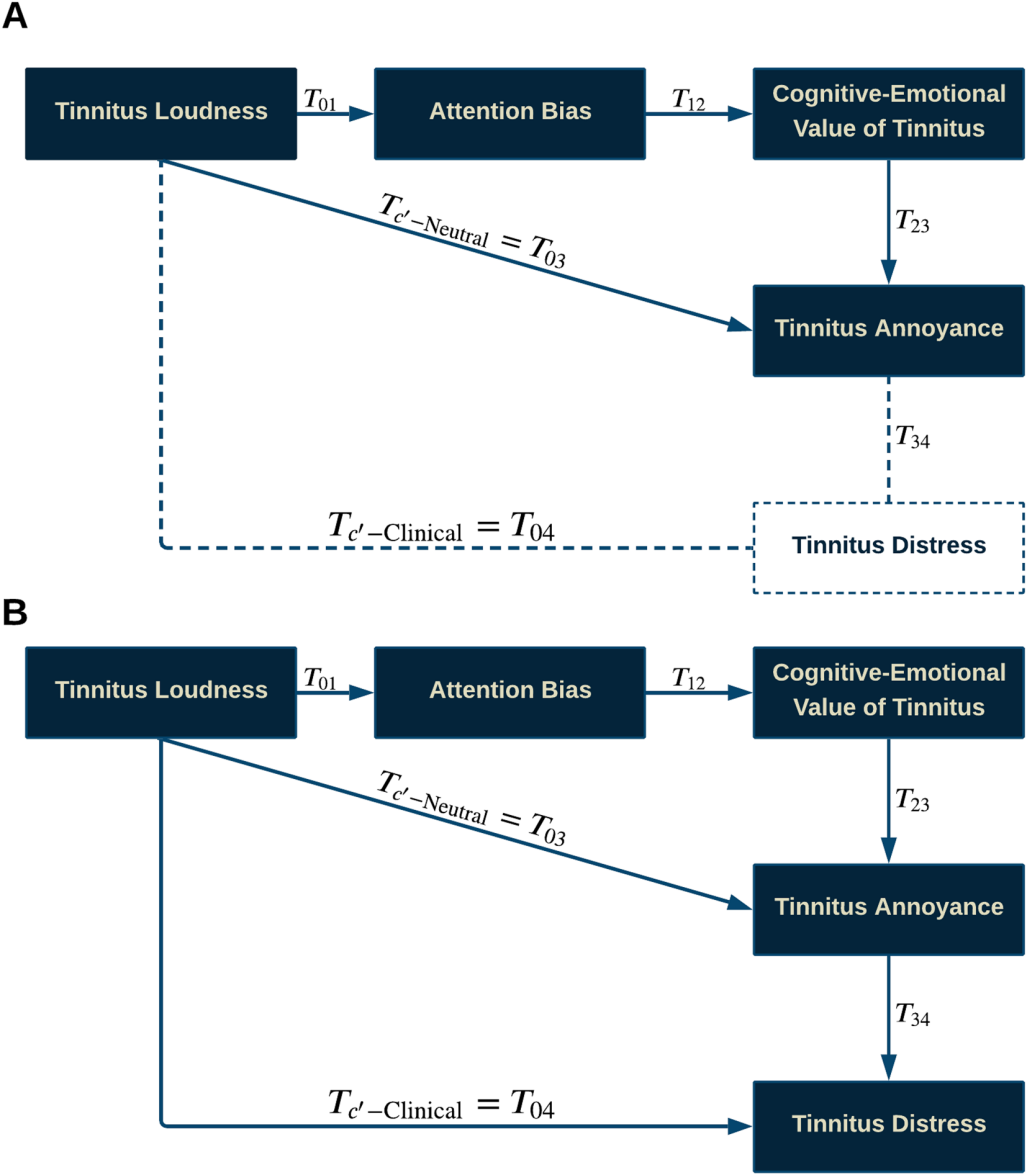
Tinnitus multi-mediator model. **A. Neutral model)** Tinnitus Loudness can lead to annoyance directly or indirectly from the *perpetual-learning process* (“${\underset{{\mathcal {N}}}{\textit{Ind-1}}}$”) that origins at the Neutral stage but always plays a principal role in the distress level. “${\underset{{\mathcal {N}}}{\textit{Ind-1}}}$”: Tinnitus Loudness $\rightarrow$ Attention bias $\rightarrow$ Cognitive-emotional value $\rightarrow$ Annoyance. **B. Clinical Model)** Tinnitus Loudness leads to distress indirectly via experiencing annoyance with or without *perpetual-learning process*. “${\underset{{\mathcal {C}}}{\textit{Ind-1}}}$”: Tinnitus Loudness $\rightarrow$ Attention Bias $\rightarrow$ Cognitive-Emotional Value $\rightarrow$ Annoyance $\rightarrow$ Clinical Distress. “${\underset{{\mathcal {C}}}{\textit{Ind-2}}}$”: Tinnitus Loudness $\rightarrow$ Annoyance $\rightarrow$ Clinical Distress.

### Statistical analysis

We employed *Durbin-Watson* to test multicollinearity-autocorrelation between independent variables in each dataset segment. Results exhibited independence in residual. Figures 3 and 4 illustrate the correlation matrices of the variables to the mediator model^20^ of the full and segmented (Neutral and Clinical) datasets, respectively.

For data analysis, *SPSS v.26* and *PROCESS* macro^48^ were employed. We customized all models with ‘10,000’ bias-corrected bootstrap samples and the fixed random-seed ‘12020’. The confidence level was chosen at 95% with ($p<$ 0.05) significance. Multiple mediation models were developed to determine the mediating effects of tinnitus-related cognitive and emotional factors in annoyance and clinical distress. Through hierarchical regression analyses, we investigated the evidence for tinnitus Neutral and Clinical CCF within the data segments. *PROCESS* macro computed standard errors, p-values, confidence intervals for direct effects, and bootstrap confidence intervals for conditional indirect effects.

### Proposed tinnitus causality (multi-mediator) model

Fundamental ideas and postulations of the multi-Mediator model:

- Tinnitus loudness represents the CAAP of the tinnitus sound.
- Failure to ignore tinnitus describes attention bias to tinnitus sound.
- Cognitive-emotional value is considered a unified parameter.
- The used dataset does not support measuring factors related to the distorted perception of tinnitus.

The causality model of neutral tinnitus explores the perpetual-learning process considering the role of evaluative conditional learning and negative appraisal (thought), which we coined as “cognitive-emotional evaluation.” The CAAP of tinnitus sound drives attention bias and subsequently triggers the cognitive-emotional evaluation of the perceived sound, leading to annoyance. However, in the Neutral stage, merely tinnitus CAAP is incapable of generating annoyance. The perpetual-learning process plays a crucial role in the transition from the Neutral to the Clinical stage. The neutral tinnitus model illustrated in (Fig. 5A) .

The clinical tinnitus causality model aims to show that clinical distress and handicap depend on the frequent experience of tinnitus-related annoyance. However, in the Clinical stage, tinnitus-related annoyance arises either through the perpetual-learning process or instantly after CAAP of tinnitus. Consequently, because of the accumulative characteristic of the ECL and appraisal in the extreme Clinical stage, the CAAP of louder tinnitus leads to a more negative cognitive-emotional value resulting in severe tinnitus-related annoyance, reinforcing the distress. The clinical tinnitus model is exhibited in (Fig. 5B)

Higher distress levels distort perception leading to louder tinnitus CAAP, which is out of the scope of this model.

## Results

### Neutral multi-mediation model of tinnitus

Multi-mediation regression analysis with the conventional least-squares method demonstrated that tinnitus loudness (CAAP) could lead to annoyance through either direct path or cascade mediators from attention bias to cognitive-emotional value. The 95% confidence interval of bootstrap results of “${\underset{{\mathcal {N}}}{{{\varvec{Ind-1}}}}}$”;$[T_{01} \times T_{12}\times T_{23}]$ revealed significantly different from zero , (0.297; between 0.2 and 0.42) in the Full-dataset, (0.24; between 0.11 and 0.42) in the Neutral-dataset, and (0.18; between 0.10 and 0.30) in the Clinical-dataset.

There was no substantial evidence within the Neutral-dataset to show tinnitus loudness $( T_{C'\text {-Neutral}}=T_{03}=0.35$, *P*-Value=0.37) from a direct path that might lead to tinnitus annoyance. However, the direct path from tinnitus loudness to annoyance in Full-dataset and Clinical-dataset were significant. As shown in Tables 2, 3, and 4 in the full-dataset, Neutral dataset, and Clinical dataset, respectively.

**Table 2:**
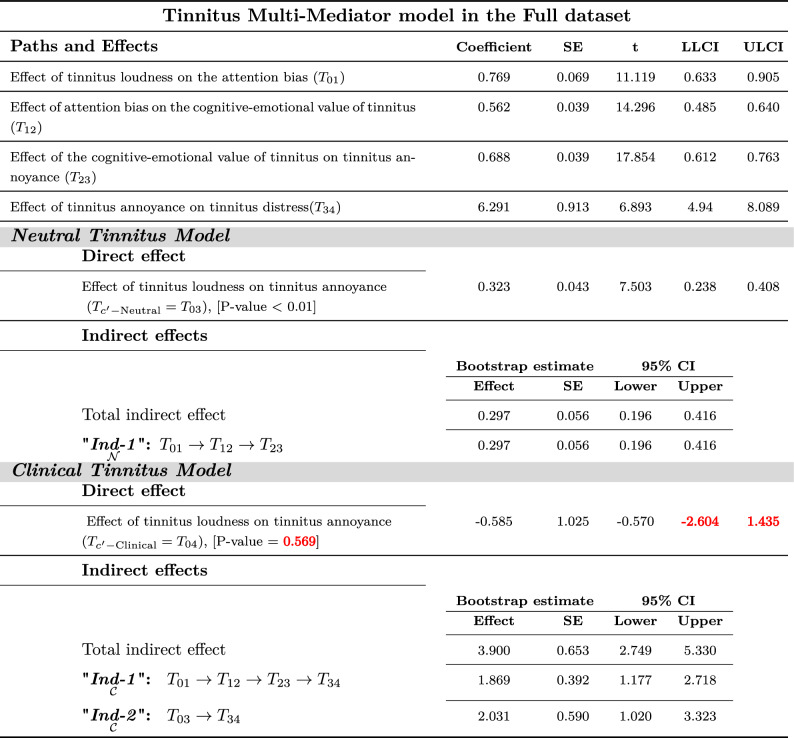
Tinnitus multi-mediator model in the Full dataset^50^ **Neutral Model**) Tinnitus Loudness can lead to annoyance directly or indirectly from *perpetual-learning process*(“${\underset{{\mathcal {N}}}{\textit{Ind-1}}}$”) that origins at the Neutral stage but always plays a principal role in the distress level. “${\underset{{\mathcal {N}}}{\textit{Ind-1}}}$”: Tinnitus Loudness $\rightarrow$ Attention bias $\rightarrow$ Cognitive-emotional value $\rightarrow$ Annoyance. **Clinical Model)** Tinnitus Loudness leads to distress indirectly via experiencing annoyance with or without *perpetual-learning process*. “${\underset{{\mathcal {C}}}{\textit{Ind-1}}}$”: Tinnitus Loudness $\rightarrow$ Attention Bias $\rightarrow$ Cognitive-Emotional Value $\rightarrow$ Annoyance $\rightarrow$ Clinical Distress. “${\underset{{\mathcal {C}}}{\textit{Ind-2}}}$”: Tinnitus Loudness $\rightarrow$ Annoyance $\rightarrow$ Clinical Distress.

Values in a similar sign (both together should be positive or both together should be negative) are considered significant.

Red color font values are insignificant.

**Table 3:**
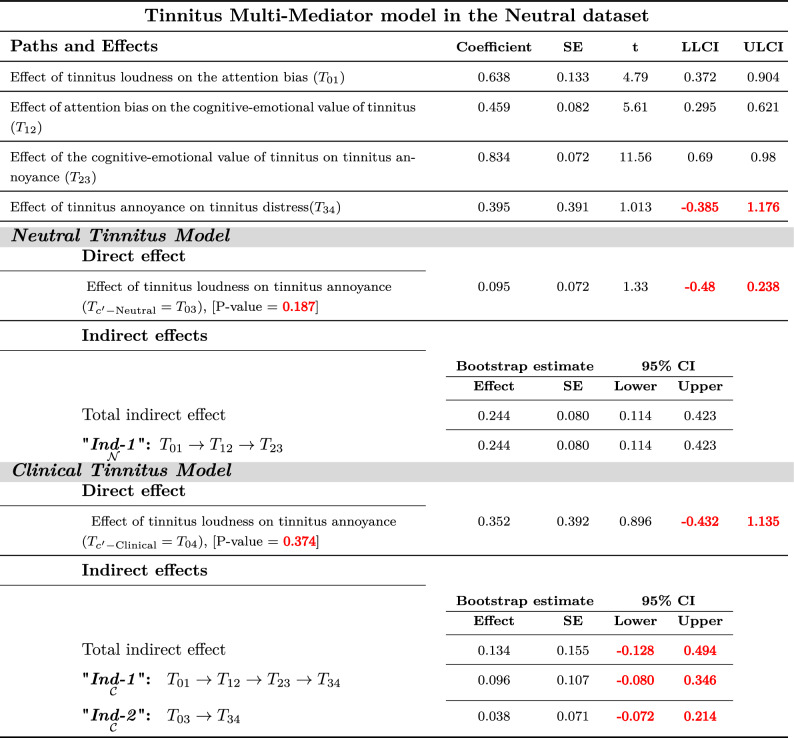
Tinnitus Multi-Mediator model in the Neutral dataset^50^: **Neutral Model)** Tinnitus Loudness can only lead to annoyance indirectly from *perpetual-learning process*(“${\underset{{\mathcal {N}}}{\textit{Ind-1}}}$”) that origins at the Neutral stage but always plays a principal role in the distress level. “${\underset{{\mathcal {N}}}{\textit{Ind-1}}}$”: Tinnitus Loudness $\rightarrow$ Attention bias $\rightarrow$ Cognitive-emotional value $\rightarrow$ Annoyance. **Clinical Model)** Tinnitus Loudness can not leads to distress either indirectly via experiencing annoyance with or without *perpetual-learning process*. “${\underset{{\mathcal {C}}}{\textit{Ind-1}}}$”: Tinnitus Loudness $\rightarrow$ Attention Bias $\rightarrow$ Cognitive-Emotional Value $\rightarrow$ Annoyance $\rightarrow$ Clinical Distress. “${\underset{{\mathcal {C}}}{\textit{Ind-2}}}$”: Tinnitus Loudness $\rightarrow$ Annoyance $\rightarrow$ Clinical Distress.

Values in a similar sign (both together should be positive or both together should be negative) are considered significant.

Red color font values are insignificant.

**Table 4:**
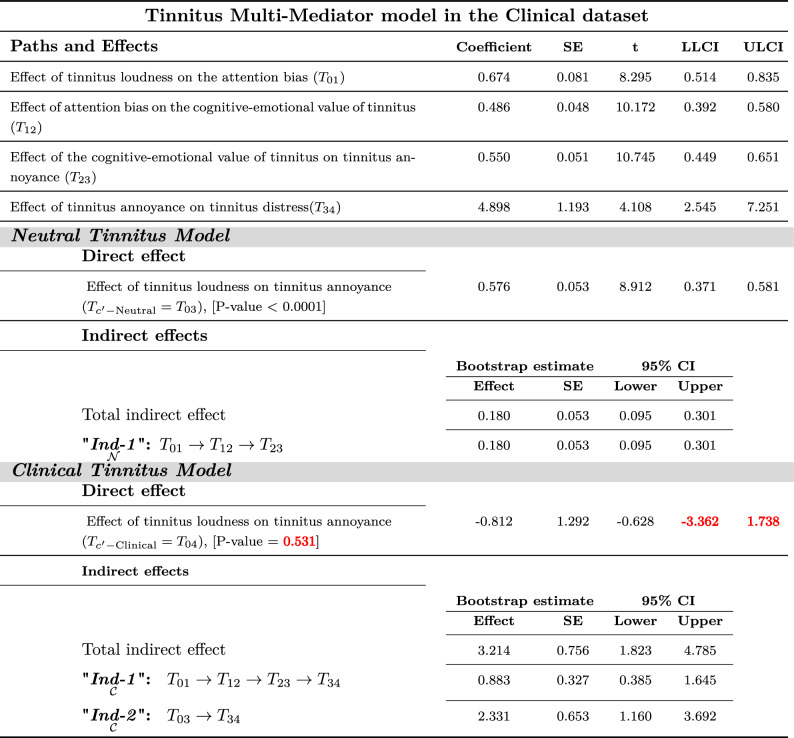
Tinnitus multi-mediator in the clinical dataset^50^; **Neutral Model)** Tinnitus Loudness can lead to annoyance directly or indirectly from *perpetual-learning process* (“${\underset{{\mathcal {N}}}{\textit{Ind-1}}}$”) that origins at the Neutral stage but always plays a principal role in the distress level. “${\underset{{\mathcal {N}}}{\textit{Ind-1}}}$”: Tinnitus Loudness $\rightarrow$ Attention bias $\rightarrow$ Cognitive-emotional value $\rightarrow$ Annoyance. **Clinical Model)** Tinnitus Loudness leads to distress indirectly via experiencing annoyance with or without *perpetual-learning process*. “${\underset{{\mathcal {C}}}{\textit{Ind-1}}}$”: Tinnitus Loudness $\rightarrow$ Attention Bias $\rightarrow$ Cognitive-Emotional Value $\rightarrow$ Annoyance $\rightarrow$ Clinical Distress. “${\underset{{\mathcal {C}}}{\textit{Ind-2}}}$”: Tinnitus Loudness $\rightarrow$ Annoyance $\rightarrow$ Clinical Distress.

Values in a similar sign (both together should be positive or both together should be negative) are considered significant.

Red color font values are insignificant.

### Clinical multi-mediation model of tinnitus

Multi-mediation regression analysis with the conventional least-squares method revealed that tinnitus loudness (CAAP) generates distress through either direct path or cascade mediators from attention bias to cognitive-emotional value leading to annoyance. The 95% confidence interval of bootstrap results of “${\underset{{\mathcal {C}}}{{{\varvec{Ind-1}}}}}$”;$[T_{01}\times T_{12}\times T_{23}\times T_{34}]$ revealed significantly different from zero (1.87; between 1.18 and 2.72) in Full-dataset and (0.88; between 0.39 and 1.65) in Clinical-dataset.

Moreover, “${\underset{{\mathcal {C}}}{{{\varvec{Ind-2}}}}}$”;$[(T_{c'-\text {Neutral}}={T_{03}})\times T_{34}]$ showed a significant difference from zero (2.03; between 1.02 and 3.32) in Full-dataset and (2.33; between 1.16 and 3.69) in Clinical-dataset. However, both the “${\underset{{\mathcal {C}}}{{{\varvec{Ind-1}}}}}$” and “${\underset{{\mathcal {C}}}{{{\varvec{Ind-2}}}}}$” were insignificant in Neutral-dataset.

No remarkable evidence was found to show tinnitus loudness from a direct path ( $T_{c'-\text {Clinical}}=T_{04}$) leading to tinnitus distress on tested datasets^50^. As shown in Tables 2, 3, and 4 in the full-dataset, Neutral dataset, and Clinical dataset, respectively.

## Clinical implications

Neurofunctional Tinnitus Model recommended that the CAAP of tinnitus is crucial for causing distress and denoted tinnitus patients into the Neutral and Clinical stages with subgroups considering mainly neuroimaging evidence. It still needs to be more explicit to suggest causal interventions. Hence, the need for formulating a conceptual and concurrently pragmatic framework toward an individualized approach for people suffering from tinnitus.

**Figure 6:**
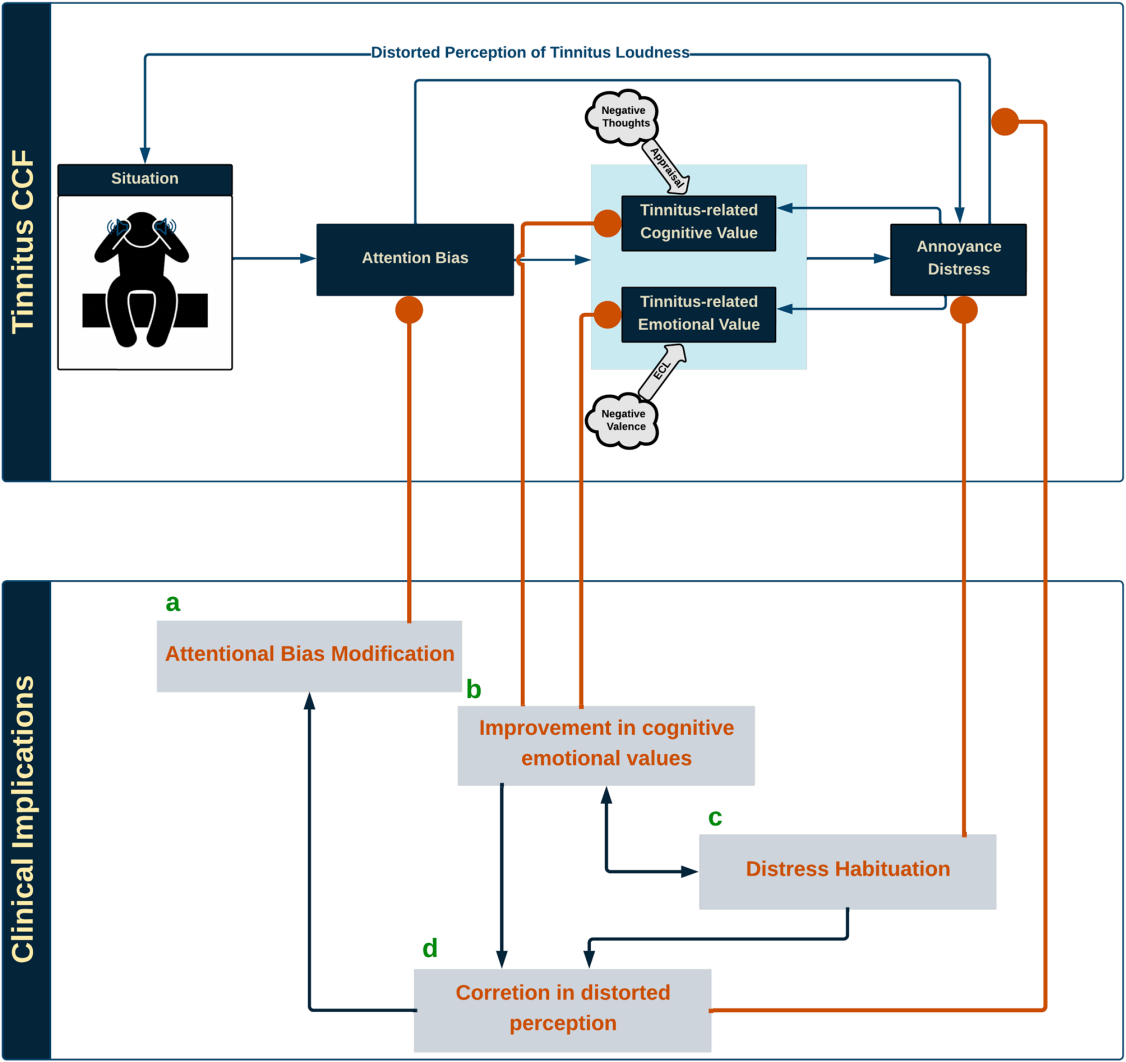
The testable framework of Clinical Implications. (**a**) “Attentional Bias Modification” delivers instant and temporary relief from symptoms at severe clinical stages. The effectiveness of other implications could indirectly influence it through corrected perceived measure factor; (**b**) “Improvement in Cognitive and Emotional values” relies on interventions that modulate tinnitus-related negative valence and arousal into neutral and positive ones, relieving annoyance and improving distorted perception measure factors. (**c**) “Distress Habituation” holds interventions that support patients in accepting possible causes of annoyance and adapting to distress with fewer reactions. It also rectifies distorted perceptions and is affected by annoyance measure factor modulation. (**d**) “ Correction in Distorted Perception” uses strategies to accurately validate the perceiving factors and feedback as a reference for correction. Good practice of clinical interventions may impact perception quality and lead to diminishing attention bias.

The proposed Conceptual Cognitive Framework, together with the tinnitus multi-mediation model, explained the detailed contribution of cognitive processes to developing and maintaining clinical tinnitus. Clinical interventions for tinnitus rehabilitation should be applied within causal and testable target-oriented implications, illustrated in Fig. 6.

The CCF proposes the following predictions in target-oriented clinical implications.

We categorized implications into **a**) “***Attentional Bias Modification***” provides instant and temporary relief from symptoms at severe clinical stages. The effectiveness of other implications could indirectly influence it via perception modification; indeed, reduction in the attention bias measure factor considers good clinical practice.

**b**)“***Improvement in Cognitive and Emotional values***” incorporates interventions that modulate tinnitus-related negative arousal (cognitive) and emotion (valence) values into neutral and positive ones, easing annoyance and improving distorted perception measure factors.

**c**) “***Distress Habituation***” contains interventions that support patients in accepting possible causes of annoyance and adapting to distress with narrower reactions. It also rectifies distorted perceptions and is affected by annoyance measure factor modulation.

**d**) “ ***Correction in Distorted Perception***” uses clinical procedures to accurately validate the perceiving factors and feedback as a reference for correction. The clinical implications mentioned earlier may impact perception quality and may lead to diminishing attention bias.

### Attentional bias modification

***Conceptual Cognitive Framework*** **predicts that attention-distraction techniques can deviate attention from tinnitus sound and prevent annoyance.**

Attention Bias Modification trains attention avoidance toward threat-related cues and decreases attention bias^52^. Several studies trained patients to shift their attention away from tinnitus toward music^53,54^, bodily sensation^55^, and positive images^56^ to reduce tinnitus distress. Similarly, relaxation training instructs patients to associate tinnitus with pleasant and relaxing mental images^57^. In tinnitus retraining therapy^58^, noise generators assist attention-shifting from an inner sound to an outside sound to help the patients cope with tinnitus. Eysel-Gosepath *et al.*^53^ investigated the effectiveness of different attention diversion forms in tinnitus therapy. Forty chronic tinnitus patients, who had received proper counseling and relaxation training, were asked to direct their attention away from tinnitus through a) sound or music; and b) imagination with light and warmth stimuli. Patients of both groups reported less annoyance and disability by tinnitus instantly after therapy and after six months^53^.

These findings suggest that tinnitus patients could use auditory and visual-thermal sensations for attention distraction purposes. However, Henry and Wilson^56^ reported that attention-switching and mental imagery exercises combined with cognitive reconstruction techniques significantly reduced tinnitus distress more than two single treatments or waiting list control^56^.

### Improvement in cognitive and emotional values

Conceptual Cognitive Frameworkdraws attention to the critical role of appraisal and emotion regulation mechanisms in reducing tinnitus-related negative cognitive and emotional values, respectively. Therefore, therapies aiming at ECL and appraisal mechanisms could reduce annoyance and distress reactions.

***Conceptual Cognitive Framework*** **predicts using Positive emotion induction techniques paired with CAAP of tinnitus could modify tinnitus-related negative valence into neutral.**

Based on the ECL mechanism, pairing CAAP of tinnitus sound with positive emotion-inducing stimuli such as pictures, films^59^, audio^60^, music, and video clips^61,62^ might reduce the negative valence of tinnitus resulting in less annoyance and minor the appearance of negative distress-reactions. Recently, Ghodratitoostani *et al.*^16^ designed an adaptive seamless observational crossover study that utilized positive emotion induction as an active control. They revealed the weakening of the negative valence of tinnitus in the Clinical-Distress stage by pairing its conscious perception with positively valenced pictures, even in the pilot study^16^. Emotion-inducing stimuli could also be presented through Game-like applications via the head-mounted display of mixed reality or smartphone screen to provide affordable home-care individualized treatments.

***Conceptual Cognitive Framework*** **predicts CBT oriented intervention is capable of declining tinnitus-related negative cognitive value**.

Cognitive Behavioural Therapy rests on the notion that our thoughts or beliefs influence our emotional and behavioral responses giving rise to cognitive, behavioral, or somatic symptoms. Cognitive behavior therapists help patients find the link between thoughts and feelings arising from an event (tinnitus) and modify their negative thoughts using education, attention manipulations, cognitive restructuring, relaxation techniques, and exposure to fearful situations^63^. Several systematic reviews and meta-analyses support the effectiveness of CBT for tinnitus. In a Cochrane approach review^47^, it was found that CBT significantly improved quality of life and decreased global tinnitus severity compared with other interventions or waiting list control conditions. Also, Hesser *et al.*^64^ conducted a meta-analysis of randomized controlled trials of CBT for tinnitus distress and reported that CBT was significantly more effective on tinnitus-related distress than active and passive control conditions. Results also showed that improvements remained over a follow-up period^64^. The results of a more newly published systematic review^65^ conform with previous studies confirming that CBT is an effective tinnitus therapy.

### Distress habituation

***Conceptual Cognitive Framework*** **predicts that Mindfulness-Based Cognitive Therapy (MBCT) reduces the negative cognitive and emotional value related to tinnitus.**

The third wave of CBT, mostly centered on acceptance and mindfulness, has recently attracted much attention. In Mindfulness-based interventions (MBIs), rather than changing the negative thoughts, patients learn to take a non-judgmental viewpoint toward their thoughts and emotions, openly attending to present-moment experiences and maintain this attention over time^66^.

Rademaker *et al.*^67^ conducted a systematic review of the MBI effect on tinnitus distress and reported that MBI could decrease the tinnitus distress score while not affecting depression anxiety in tinnitus patients. Using a qualitative approach focused on the individuals’ experiences, Marks *et al.*^68^ investigated how and why MBCT could reduce tinnitus-related distress. They revealed that multiple processes, including mindful awareness, attitudes of equanimity, kindness, and compassion, helped patients change their relationship with tinnitus from fighting with it to accepting it as it is^68^. In a pilot study, Husain *et al.*^69^ also revealed that MBCT is a sufficient clinical implication for treating distressing tinnitus based on neuroanatomical changes reflecting reductions in tinnitus-related severity.

### Correction in distorted perception

***Conceptual Cognitive Framework*** **predicts technology-based approaches to provide the online measurement of tinnitus loudness-match can render to correct distorted perception in loudness judgment.**

Recently, Probst *et al.*^23^ utilized a mobile application to track changes in rating tinnitus loudness in daily life to indicate what can distress patients. He reported that emotional state partially mediates the relationship between tinnitus loudness and tinnitus distress.

***Conceptual Cognitive Framework*** **predicts clinical interventions that endeavor to modulate or regulate tinnitus-related cognitive-emotional values, including CBT, MBCT could decrease the experience of tinnitus distress and prevent the distorted perception of tinnitus loudness.**

McKenna *et al.*^70^ conducted a randomized controlled study MBCT to treat chronic tinnitus and reported a significant reduction in the self-report tinnitus loudness perception.

***Conceptual Cognitive Framework*** **predicts neuromodulation techniques to improve the brain’s emotion regulatory function can cause a correction in the distorted perception of tinnitus loudness.**

transcranial Direct Current Stimulation over the brain (dlPFC) provided promising results in tinnitus-distress reduction as determined with visual analog scale and numerical rating scale^71–73^. Shekhawat and Vanneste^74^ used HD-tDCS with anode as the central electrode placed on the right-dlPFC and reported a significant reduction in tinnitus loudness but not annoyance. Recently, Ghodratitoostani *et al.*^16^ demonstrated a well-controlled dose-response study of anodal HD-tDCS on the left-dlPFC concurrent with the presentation of positively valenced pictures to affect positive emotion network underneath mounted electrodes. Reported preliminary results were promising to illuminate the role of emotion regulation in tinnitus loudness perception^16^. Inconsistent findings highlight the need for further research and studies to explore the predictions mentioned earlier precisely.

## Future trends

The clinical recommendations provided in this paper can be applied separately or in combination to plan treatment and prevention based on the clinical and neutral stages, respectively. The CCF builds upon the general assumption that patients should be consciously and actively involved in rehabilitation. Subsequently, new treatments can be developed aimed at encouraging patients to be consciously aware of their tinnitus and contingencies of the induced positive emotion for intervention. Likewise, tinnitus is a complex condition influenced by social cognition, including social and cultural factors^75,76^, that can affect annoyance and distress levels. Therefore, it is helpful to consider sociocultural factors in future individualized clinical interventions. furthermore, surrogate measurements are recommended to guarantee the patient’s conscious attended awareness.

The CCF can provide a decision-support platform for clinicians to deliver causal target-oriented interventions. Eventually, the methodologies suggested can provide a reliable platform to build a CCF for other cognitive disorders besides complex comorbidities and support the causal clinical data models. For instance, insomnia and sleep deprivation can facilitate the perpetual-learning process of tinnitus. Considering the recently projected CCF for Insomnia CCF^77^ and mini-review in tinnitus-insomnia comorbidity^78^, we can propose further investigations on the future advanced causal clinical decision-making incorporates tinnitus and insomnia together. This approach may also improve our knowledge of psychological disorders and complicated comorbidities by supporting the design of different clinical recommendations for cognitive rehabilitation^18,79^ and demonstrating comprehensive frameworks in line with the “preventive medicine” policy.

## Limitations

The CCF of tinnitus, its predictions, and the corresponding suggested interventions do not include patients with general cognitive distortion and psychotic problems. Large-scale repeated measures and well-controlled randomized longitudinal studies such as dose-response relationships are required to improve causal predictability and be capable of evaluating interventions’ efficacy.

### Ethical approval and consent to participate

Data were collected from participants of two running studies on neurofunctional tinnitus model validation—(1) A randomized crossover three-session double-blind study and (2) An observational prospective cohort study, both approved by the Ethics Committee for Analysis of Research Projects, Specialized Center of Otorhinolaryngology and Speech Therapy, Hospital das Clínicas de Ribeirão Preto, University of São Paulo, Brazil (HCRP No. 55716616.1.1001.5440, and HCRP No. 09813519.1.0000.5440; internationally registered with U1111-1236-5441). All subjects gave written informed consent.

### Supplementary Information

Supplementary Information.

## Data Availability

Datasets, analyses, and related syntax used in this study are available from the corresponding author on reasonable request by filling out the NEL-Consent^50^. Technical Report was also published^20^ to provide the simplified theoretical knowledge needed to interpret and develop new mediatory models by non-experts in statistical modeling for cognitive problems.

## Abbreviations

CAAP: Continuous attended awareness perception
CBT: Cognitive behavioural therapy
CCF: Conceptual cognitive framework
CS: Conditional stimulus
ECL: Evaluative conditional learning
EST: Emotional stroop task
HD: High definition
MBCT: Mindfulness-based cognitive therapy
MBI: Mindfulness-based interventions
NfTM: Neurofunctional tinnitus model
tDCS: Transcranial direct current stimulation
THI: Tinnitus handicap inventory
TS: Tinnitus severity
US: Unconditional stimulus
