# Supplementary material for: Conceptual framework for tinnitus: a cognitive model in practice
Source: Sci Rep. 2024 Mar 26;14:7186. doi: 10.1038/s41598-023-48006-7 (PMC10965907; doi:10.1038/s41598-023-48006-7)
Supplement: Supplementary file 1 — Supplementary Information. [file 41598_2023_48006_MOESM1_ESM.pdf]

Supplementary Material for Conceptual Framework for Tinnitus

Iman Ghodratitoostani [1,3*], Zahra Vaziri [1,2], Milton Miranda Neto [1,3], Camila de Giacomo Carneiro [1,4], Alexandre Claudio Botazzo Delbem [1,3], Miguel Angelo Hyppolito [5], Hamid Jalilvand [6], Francisco Louzada [3], and and Joao Pereira Leite [2]

[1]Neurocognitive Engineering Laboratory (NEL), Center for Engineering Applied to Health, Institute of Mathematics and Computer Science, University of Sao Paulo, Brazil; [2]Department of Neurosciences and Behavioral Sciences, Medical School of Ribeirão Preto, University of São Paulo, Brazil; [3]Institute of Mathematics and Computer Science, University of São Paulo, São Carlos, Brazil; [4]Department of Otorhinolaryngology, Ribeirão Preto Medical School, Universidade de São Paulo, Ribeirão Preto, Brazil; [5]Department of Ophthalmology, Otorhinolaryngology, Head and Neck Surgery, Ribeirão Preto Medical School, University of São Paulo, Brazil; [6]Department of Audiology, School of Rehabilitation, Shahid Beheshti University of Medical Sciences, Tehran, Iran;

Supplementary Material for Conceptual Framework for Tinnitus

Supplementary Table 1

We depicted Tinnitus Models that have been suggested with the corresponding collections of clinical evidence.

Investigator	Model Definition	Clinical Evidence
**Hallam** *et al.* **(1984)**	proposed that failure in habituation to tinnitus causes increased awareness because of negative appraisal and emotional significance.	Most tinnitus experiences do not complain about it [18], but tinnitus sufferers more persistently perceive tinnitus than others.
		Tolerance for the tinnitus symptom is a function of time since onset Hazell (1979) and [87], and it diminishes by stress, worry, and comorbidities.
		Complaint behaviour showed no relationship with perceived loudness of tinnitus[33].
**Jastreboff** *et al.* **(1996)**	classical conditioning was proposed as the principal mechanism behind the aversive emotional states of tinnitus.	Tinnitus severity and its impact on life reveal no correlation with its psychoacoustic parameters [43, 48].
		Non-auditory systems are responsible for the tinnitus severity and annoying level.[58]
		The majority (94%) of participants of the soundproof room experiment after 5 minutes heard sounds similar to those described by tinnitus sufferers, which disappeared after they left the room [47]
		Tinnitus with normal hearing was documented in 20% of patients [21], and almost 27% of totally deafened people do not experience tinnitus [40].
		About 40% of tinnitus patients showed increased sensitivity levels to environmental sound. [42, 56]
		Tinnitus incident was reported twice in a hearing-impaired population versus normal hearing [19]. Concurrently, 20% of tinnitus sufferers have normal hearing [21], and almost 27% of totally deafened people do not experience tinnitus [43].
		No specific pattern was observed in tinnitus population audiograms.[41]
		Tinnitus is a phantom sound. [57, 74].

Continued on next page

Supplementary Table 1 – continued from previous page

Investigator	Model Definition	Clinical Evidence
		The majority of tinnitus experiences can naturally habituate to it [21, 70]. Habituation leading to ignoring tinnitus's presence since they consciously attend to tinnitus [82].
		Approximately 50% of tinnitus patients reported disturbed sleep [87].
		Tinnitus affects sleep and is particularly intense in preventing the continuation of sleep[71].
		Clinically significant tinnitus usually emerges during a period associated with external emotional stress [41].
**Zenner & Zalaman (2004)**	postulated that tinnitus sensitization develops when perceiving sound is classified as noxious, fear-inducing, unpredictable, and might cause a sense of deficiency in coping and helplessness.	Cognitive Desensitized Therapy revealed reduce in psychosomatic tinnitus burden and the daily observation time and an improvement in the quality of life [101].
		Negative thinking about tinnitus is an essential component of tinnitus distress.
		The deficiency of positive thoughts does not necessarily indicate negative thoughts in tinnitus [100].
		Tinnitus-related distress or handicap associated with tinnitus complaint reflects cognitive and behavioral aspects[36, 62].
		More catastrophic[15, 85] thoughts negatively correlated with quality of life [93] and positively correlated with tinnitus severity.
		Cognitive-behavioral therapy disrupts cycles that cause tinnitus distress [49] and reduces catastrophic thinking[96].
		Negative and catastrophic thinking is associated with emotional distress [100], depressive symptoms [97], fearful beliefs, and increased attention toward tinnitus [15].
		Tinnitus-related bothersome level boosts autonomic arousal responses [45] but delays responses to stress-induced tasks [44]. Tinnitus patients showed increased muscle tension [2, 75], which reduces by psychophysiological therapy.

Continued on next page

Supplementary Table 1 – continued from previous page

Investigator	Model Definition	Clinical Evidence
		Anxiety sensitivity significantly predicts tinnitus distress, and high levels of trait anxiety were observed among patients with tinnitus [35].
		Bothersome tinnitus exhibited that is associated with emotional distress [35, 69, 92, 107].
		Patients in the clinical tinnitus stage experience psychiatric disorders in their lifetime, particularly anxiety and depression [66, 105, 106].
**McKenna *et al.* (2014)**	proposed the Cognitive Behavioral Model of Tinnitus	Psychological therapies, specifically CBT for tinnitus, revealed a significant and sustained positive effect on tinnitus-related annoyance and distress [5, 51, 67].
		People with tinnitus are weaker in executive control of attention but not selective attention processing [4, 46, 78, 83]. Tinnitus-related annoyance was revealed to be highly correlated to self-reported inability to ignore tinnitus[52]. Patients seeking tinnitus the treatment reported being aware of their tinnitus more frequently [92].
		Negative tinnitus experience was more correlated with lower quality of life [15, 90].
		A significant positive correlation was reported between Tinnitus Vigilance and awareness [15].
		Tinnitus loudness misperception [10, 53]explored a significant relationship to catastrophizing but no change due to therapy [5].
		Optimistic illness descriptors connected with reduced levels of depression in patients with tinnitus [1, 13, 77, 91].
		Personality traits are significantly related to tinnitus distress [29, 39]and predict tinnitus discomfort [81].
		Avoidance behavior is connected to more distressing tinnitus [50], like avoiding silence due to fear of cope incapability [65, 84].
		Auditory perception occurs in the absence of auditory input. It has been shown that more than 80% of people with normal hearing can perceive phantom sounds in a soundproof room [11].

Continued on next page

Supplementary Table 1 – continued from previous page

Investigator	Model Definition	Clinical Evidence
		In perception without awareness, the subject does not consciously identify it or even detect its presence [24].
		Tinnitus perception is not just a sound percept, but it also includes affective components intimately linked to the sound percept due to the deafferentation of auditory input [8, 37].
		As a result of auditory deafferentation, the amount of information necessary to make sense of the world by the brain is limited [23].
**De Ridder *et al.* (2014)**	Proposed an integrative model of auditory phantom perception: Tinnitus as a unified percept of interacting separable subnetworks.	Loudness perception occurs due to a dysfunctional noise-suppressing mechanism that is probably limbically driven [64, 76].
		Anatomically [80] and functionally [74], the auditory cortex is altered in people with tinnitus.
		Non-auditory brain areas are changed structurally in tinnitus [88]
		Severe tinnitus may result in distress [12], anxiety[9], depression [25], cognitive dysfunction [37], insomnia [20] and to a significant decrease in the quality of life [9].
		More than an exclusive focus on the auditory cortex is required to understand tinnitus in a clinically relevant sense. We need to consider the importance of distributed networks [27].
		The cognitive disruption caused by tinnitus might be a starting point for later conditioned emotional reactions to tinnitus [3, 73].
		Cognitive inefficiency in tinnitus patients is related to the control of attentional processes [37].
**Andersson & McKenna (2006)**	A model of the road to annoyance via different levels of cognitive functioning.	There are two forms of distressed tinnitus patients: one characterized by depression and the other by anxiety [28].
		One problem with all self-report instruments, known as “negative affect”, is that they are liable to confound general distress with specific complaints [95].

Continued on next page

Supplementary Table 1 – continued from previous page

Investigator	Model Definition	Clinical Evidence
		Tinnitus often requires specialist consultation, and longitudinal data suggest that the rate of spontaneous improvement is low [7].
**Ghodratitoostani *et al.* (2016) and Ghodratitoostani *et al.* (2016)**	Neurofunctional Tinnitus Model	The bottom-up selective attention processes support the suppression of irrelevant stimuli, which may occur at the early stages by the TRN along the “lateral inhibition mechanism” [60, 104].
		Lateral inhibition ideally suppresses the noise originating from distracters and facilitates the processing essential stimuli. The amygdala, posterior orbitofrontal cortex, and mediodorsal thalamus ending at the Thalamic reticular nucleus may suppress the signal of distracting stimuli at sensory cortices [103].
		The decline of the vmPFC inhibitory output revealed a reduction in gray matter in vmPFC, leading to increased activity of NAc [79, 89].
		The relevance of stimulus requires an active associated neuronal network to indicate its corresponding value or reward outcome in a particular context, called the “self-control” process [59].
		The top-down relevant (cognitive emotional valued) signal can reach the attentive processes whereby the signal can become aware or unaware [34].
		The hippocampus structure is not necessarily involved in tinnitus generation; however, the paralimbic is involved in the generation of tinnitus [68, 89].
		Personality disorders include the potential triggers of tinnitus and available tinnitus managements and treatments leading to appraisals such as “tinnitus is detrimental to my health” or “now, I am becoming deaf” [101].
		The Neurofunctional Tinnitus Model hypothesizes that the perception of sound fundamentally depends on the allocation of attentional resources via the frontal cortex, which in turn, depends on the cognitive-emotional value and the relevance of the phantom stimulus to the context [31, 32].

Continued on next page

Supplementary Table 1 – continued from previous page

Investigator	Model Definition	Clinical Evidence
		The ability to consciously report sensory inputs is theorized as the perception process. We propose to add more details to the model of distinction between awareness and attention [63, 94].
**Cima (2018)**	Fear-avoidance model of chronic tinnitus	Analogous to observations in chronic pain research, bothersome tinnitus is considered an illness rather than a disease [30, 86].
		The negative interpretation of the signal and related heightened autonomic arousal levels would lead to dysfunctional cognitive processing and, therefore, disrupt habituation [16].
		Since the medical or pharmacological cure is unavailable [26, 27], current theoretical frameworks have been explanatory on some level. The cognitive-behavioral treatments (CBT) for tinnitus have been shown to be effective in decreasing tinnitus distress, anxiety, and annoyance and improving daily life functioning [17]. The resulting treatment approaches have alleviated complaints leading to reports of occasional recovery to a satisfactory quality of daily life in many patients.
		Conditioned fear responses elicited by the tinnitus sound cause the tinnitus becoming bothersome [55, 57].
		Mainly through negative cognitive misinterpretations of the tinnitus signal, distress and bodily arousal are provoked, leading to inaccurate evaluations of sensory activity and distorted perceptions [72].
		Individuals perceiving the tinnitus signal are subject to automatic emotional and sympathetic responses. These symptoms are misinterpreted as harmful or threatening [15, 61, 72].
		The merits of Cognitive Behavioural Treatments (CBT) for tinnitus have decreased tinnitus disability and distress for a large group of patients [50, 54].
		Fear-related safety-seeking behaviors were postulated to be crucial in explaining increased suffering in tinnitus patients. Evidence revealed that the tendency to avoid so-called ‘unsafe’ stimuli or events because of the tinnitus mediates the association between tinnitus severity and quality of life [98, 99].

Continued on next page

Supplementary Table 1 – continued from previous page

Investigator	Model Definition	Clinical Evidence
		The fear of bodily sensations was strongly related to tinnitus distress, again fully mediated by tinnitus-related avoidance behaviours [50].
		A fear-avoidance model for bothersome tinnitus [15, 61] offers predictions about the behavioral components in the maintenance of tinnitus- disability.

References

1. Andersson, G. The role of optimism in patients with tinnitus and in patients with hearing impairment. *Psychology and Health* **11,** 697–707 (1996).

2. Andersson, G., &dagger, Lyttkens, L. & Larsen, H. Distinguishing levels of tinnitus distress. *Clinical Otolaryngology & Allied Sciences* **24,** 404–410 (1999).

3. Andersson, G., Baguley, D., McKenna, L. & McFerran, D. *Tinnitus: A multidisciplinary approach* (Whurr, 2005).

4. Andersson, G., Eriksson, J., Lundh, L.-G. & Lyttkens, L. Tinnitus and cognitive interference: a Stroop paradigm study. *Journal of Speech, Language, and Hearing Research* **43,** 1168–1173 (2000).

5. Andersson, G. & Lyttkens, L. A meta-analytic review of psychological treatments for tinnitus. *British journal of audiology* **33,** 201–210 (1999).

6. Andersson, G. & McKenna, L. The role of cognition in tinnitus. *Acta Oto-Laryngologica* **126,** 39–43 (2006).

7. Andersson, G., Strömgren, T., Ström, L. & Lyttkens, L. Randomized controlled trial of internet-based cognitive behavior therapy for distress associated with tinnitus. *Psychosomatic medicine* **64,** 810–816 (2002).

8. Axelsson, A. & Ringdahl, A. Tinnitus—a study of its prevalence and characteristics. *British journal of audiology* **23,** 53–62 (1989).

9. Bartels, H., Middel, B. L., van der Laan, B. F., Staal, M. J. & Albers, F. W. The additive effect of co-occurring anxiety and depression on health status, quality of life and coping strategies in help-seeking tinnitus sufferers. *Ear and hearing* **29,** 947–956 (2008).

10. Basile, C. É., Fournier, P., Hutchins, S. & Hébert, S. Psychoacoustic assessment to improve tinnitus diagnosis. *PloS one* **8,** e82995 (2013).

11. Bo, L. D. *et al.* Tinnitus aurium in persons with normal hearing: 55 years later. *Otolaryngology–Head and Neck Surgery* **139,** 391–394 (2008).

12. Budd, R. J. & Pugh, R. Tinnitus coping style and its relationship to tinnitus severity and emotional distress. *Journal of psychosomatic research* **41,** 327–335 (1996).

13. Buysse, D. J. *et al.* Diagnostic concordance for DSM-IV sleep disorders: a report from the APA/NIMH DSM-IV field trial. *The American journal of psychiatry* (1994).

14. Cima, R. Bothersome tinnitus. *Hno* **66,** 369–374 (2018).

15. Cima, R. F., Crombez, G. & Vlaeyen, J. W. Catastrophizing and fear of tinnitus predict quality of life in patients with chronic tinnitus. *Ear and hearing* **32,** 634–641 (2011).

16. Cima, R. F., van Breukelen, G. & Vlaeyen, J. W. Tinnitus-related fear: Mediating the effects of a cognitive behavioural specialised tinnitus treatment. *Hearing research* **358,** 86–97 (2018).

17. Cima, R. F. *et al.* Specialised treatment based on cognitive behaviour therapy versus usual care for tinnitus: a randomised controlled trial. *The Lancet* **379,** 1951–1959 (2012).

18. Coles, R., Davis, A. & Haggard, M. *Medical Research Council's Institute of Hearing Research. Epidemiology of tinnitus* in *CIBA Found Symp* **85** (1981), 16–34.

19. Coles, R. & Hallam, R. S. Tinnitus and its management. *British medical bulletin* **43,** 983–998 (1987).

20. Cronlein, T., Langguth, B., Geisler, P. & Hajak, G. Tinnitus and insomnia. *Prog Brain Res* **166,** 227–33. ISSN: 0079-6123 (Print) 0079-6123 (Linking) (2007).

21. Davis, A. & El Refaie, A. Epidemiology of tinnitus. Tinnitus handbook (Singular Audiology Text), Singular Pub. *Group* (2000).

22. De Ridder, D. *et al.* An integrative model of auditory phantom perception: tinnitus as a unified percept of interacting separable subnetworks. *Neurosci Biobehav Rev* **44,** 16–32. ISSN: 1873-7528 (Electronic) 0149-7634 (Linking) (2014).

23. De Ridder, D., Vanneste, S. & Freeman, W. The Bayesian brain: phantom percepts resolve sensory uncertainty. *Neuroscience & Biobehavioral Reviews* **44,** 4–15 (2014).

24. Dehaene, S. *et al.* Imaging unconscious semantic priming. *Nature* **395,** 597–600 (1998).

25. Dobie, R. A. Depression and tinnitus. *Otolaryngologic Clinics of North America* **36,** 383–388 (2003).

26. Elgoyhen, A. B. & Langguth, B. Pharmacological approaches to the treatment of tinnitus. *Drug discovery today* **15,** 300–305 (2010).

27. Elgoyhen, A. B., Langguth, B., Vanneste, S. & De Ridder, D. Tinnitus: network pathophysiology-network pharmacology. *Frontiers in systems neuroscience* **6,** 1 (2012).

28. Erlandsson, S. I. *Tinnitus: tolerance or threat?: psychological and psychophysiological perspectives* (1990).

29. Eysenck, H. J. A short questionnaire for the measurement of two dimensions of personality. *Journal of Applied Psychology* **42,** 14 (1958).

30. Gatchel, R. J., Peng, Y. B., Peters, M. L., Fuchs, P. N. & Turk, D. C. The biopsychosocial approach to chronic pain: scientific advances and future directions. *Psychological bulletin* **133,** 581 (2007).

31. Ghodratitoostani, I. *et al.* Theoretical Tinnitus Framework: A Neurofunctional Model. *Front Neurosci* **10,** 370. ISSN: 1662-4548 (Print) 1662-453X (Linking) (2016).

32. Ghodratitoostani, I. *et al.* Theoretical tinnitus multimodality framework: a neurofunctional model. *Journal of Advanced Medical Sciences and Applied Technologies* **2,** 181–189 (2016).

33. Goodwin, P. E. & Johnson, R. M. The loudness of tinnitus. *Acta oto-laryngologica* **90,** 353–359 (1980).

34. Graziano, M. S. & Webb, T. W. The attention schema theory: a mechanistic account of subjective awareness. *Front Psychol* **6,** 500. ISSN: 1664-1078 (Electronic) 1664-1078 (Linking) (2015).

35. Halford, J. B. & Anderson, S. D. Anxiety and depression in tinnitus sufferers. *Journal of psychosomatic research* **35,** 383–390 (1991).

36. Hallam, R. S. Correlates of sleep disturbance in chronic distressing tinnitus. *Scand Audiol* **25,** 263–6. ISSN: 0105-0397 (Print) 0105-0397 (Linking) (1996).

37. Hallam, R., McKenna, L. & Shurlock, L. Tinnitus impairs cognitive efficiency. *International journal of audiology* **43,** 218–226 (2004).

38. Hallam, R., Rachman, S. & Hinchcliffe, R. Psychological aspects of tinnitus. *Contributions to medical psychology* **3,** 31–53 (1984).

39. Hathaway, S. R. & McKinley, J. C. A multiphasic personality schedule (Minnesota): I. Construction of the schedule. *The Journal of Psychology* **10,** 249–254 (1940).

40. Hazell, J., McKinney, C. & Aleksy, W. Mechanisms of tinnitus in profound deafness. *The Annals of otology, rhinology & laryngology. Supplement* **166,** 418–420 (1995).

41. Hazell, J. *Support for neurophysiological model of tinnitus* in *Proceedings of the fifth international tinnitus seminar* (1996).

42. Hazell, J., von Schoenberg, L., Meerton, L. & Sheldrake, J. *Tinnitus and the unilateral dead ear* in *Tinnitus 91. Proceedings of the Fourth International Tinnitus Seminar* (1992), 261–264.

43. Hazell, J. *et al.* A clinical study of tinnitus maskers. *British Journal of Audiology* **19,** 65–146 (1985).

44. Hébert, S. & Lupien, S. J. The sound of stress: blunted cortisol reactivity to psychosocial stress in tinnitus sufferers. *Neuroscience letters* **411,** 138–142 (2007).

45. Hébert, S., Paiement, P. & Lupien, S. J. A physiological correlate for the intolerance to both internal and external sounds. *Hearing research* **190,** 1–9 (2004).

46. Heeren, A. *et al.* Tinnitus specifically alters the top-down executive control sub-component of attention: evidence from the attention network task. *Behavioural brain research* **269,** 147–154 (2014).

47. Heller, M. F. & Bergman, M. VII Tinnitus aurium in normally hearing persons. *Annals of Otology, Rhinology & Laryngology* **62,** 73–83 (1953).

48. Henry, J. A. & Meikle, M. B. Psychoacoustic measures of tinnitus. *Journal of the American Academy of Audiology* **11** (2000).

49. Henry, J. & Wilson, P. The psychological management of tinnitus: comparison of a combined cognitive educational program, education alone and a waiting-list control. *The international tinnitus journal* **2,** 9–20 (1996).

50. Hesser, H. & Andersson, G. The role of anxiety sensitivity and behavioral avoidance in tinnitus disability. *International Journal of Audiology* **48,** 295–299 (2009).

51. Hesser, H., Weise, C., Westin, V. Z. & Andersson, G. A systematic review and meta-analysis of randomized controlled trials of cognitive–behavioral therapy for tinnitus distress. *Clinical psychology review* **31,** 545–553. ISSN: 0272-7358 (2011).

52. Hiller, W. & Goebel, G. When tinnitus loudness and annoyance are discrepant: audiological characteristics and psychological profile. *Audiology and Neurotology* **12,** 391–400 (2007).

53. Hoare, D. J., Kowalkowski, V. L. & Hall, D. A. Effects of frequency discrimination training on tinnitus: results from two randomised controlled trials. *Journal of the Association for Research in Otolaryngology* **13,** 543–559 (2012).

54. Hoare, D. J., Kowalkowski, V. L., Kang, S. & Hall, D. A. Systematic review and meta-analyses of randomized controlled trials examining tinnitus management. *The Laryngoscope* **121,** 1555–1564 (2011).

55. Jastreboff, P. J. Phantom auditory perception (tinnitus): mechanisms of generation and perception. *Neuroscience research* **8,** 221–254 (1990).

56. Jastreboff, P. J., Gray, W. C., Gold, S. L., *et al.* Neurophysiological approach to tinnitus patients. *American Journal of Otology* **17,** 236–240 (1996).

57. Jastreboff, P. J. & Hazell, J. W. A neurophysiological approach to tinnitus: clinical implications. *British journal of audiology* **27,** 7–17 (1993).

58. Jastreboff, P. J., Hazell, J. W. & Graham, R. L. Neurophysiological model of tinnitus: dependence of the minimal masking level on treatment outcome. *Hearing Research* **80,** 216–232 (1994).

59. Kaping, D., Vinck, M., Hutchison, R. M., Everling, S. & Womelsdorf, T. Specific contributions of ventromedial, anterior cingulate, and lateral prefrontal cortex for attentional selection and stimulus valuation. *PLoS Biol* **9,** e1001224. ISSN: 1545-7885 (Electronic) 1544-9173 (Linking) (2011).

60. Kiang, N. Y., Moxon, E. C. & Levine, R. A. Auditory-nerve activity in cats with normal and abnormal cochleas. In: Sensorineural hearing loss. *Ciba Found Symp,* 241–73. ISSN: 0300-5208 (Print) 0300-5208 (Linking) (1970).

61. Kleinstäuber, M. *et al.* The role of fear-avoidance cognitions and behaviors in patients with chronic tinnitus. *Cognitive behaviour therapy* **42,** 84–99 (2013).

62. Kuk, F. K., Tyler, R. S., Russell, D. & Jordan, H. The psychometric properties of a tinnitus handicap questionnaire. *Ear and hearing* **11,** 434–445 (1990).

63. Lamme, V. A. Why visual attention and awareness are different. *Trends Cogn Sci* **7,** 12–18. ISSN: 1879-307X (Electronic) 1364-6613 (Linking) (2003).

64. Leaver, A. M. *et al.* Dysregulation of limbic and auditory networks in tinnitus. *Neuron* **69,** 33–43. ISSN: 1097-4199 (Electronic) 0896-6273 (Linking) (2011).

65. Mackenna, L. & Irwin, R. Sound Therapy for tinnitus–sacred cow or idol worship. *An investigation of the evidence. Audiolol med* **6,** 16–24 (2008).

66. Marciano, E. *et al.* Psychiatric comorbidity in a population of outpatients affected by tinnitus: Comorbilidad psiquiátrica en una población de pacientes de consulta externa afectados por tinnitus. *International journal of audiology* **42,** 4–9 (2003).

67. Martinez-Devesa, P., Waddell, A., Perera, R. & Theodoulou, M. Cognitive behavioural therapy for tinnitus. *Cochrane database of systematic reviews* (2007).

68. Maudoux, A. *et al.* Connectivity graph analysis of the auditory resting state network in tinnitus. *Brain Res.* ISSN: 1872-6240 (Electronic) 0006-8993 (Linking) (2012).

69. McCormack, A. *et al.* The prevalence of tinnitus and the relationship with neuroticism in a middle-aged UK population. *Journal of Psychosomatic Research* **76,** 56–60 (2014).

70. McFadden, D. *Tinnitus: Facts, theories, and treatments* (National Academy Press, 1982).

71. McKenna, K. Y. & Bargh, J. A. Plan 9 from cyberspace: The implications of the Internet for personality and social psychology. *Personality and social psychology review* **4,** 57–75 (2000).

72. McKenna, L., Handscomb, L., Hoare, D. J. & Hall, D. A. A scientific cognitive-behavioral model of tinnitus: novel conceptualizations of tinnitus distress. *Frontiers in Neurology* **5,** 196 (2014).

73. Mirz, F. *et al.* Positron emission tomography of cortical centers of tinnitus. *Hearing research* **134,** 133–144 (1999).

74. Muhlnickel, W., Elbert, T., Taub, E. & Flor, H. Reorganization of auditory cortex in tinnitus. *Proc Natl Acad Sci U S A* **95,** 10340–3. ISSN: 0027-8424 (Print) 0027-8424 (Linking) (1998).

75. Peroz, I. Dysfunctions of the stomatognathic system in tinnitus patients compared to controls. *HNO* **51,** 544–549 (2003).

76. Rauschecker, J. P., Leaver, A. M. & Muhlau, M. Tuning out the noise: limbic-auditory interactions in tinnitus. *Neuron* **66,** 819–26. ISSN: 1097-4199 (Electronic) 0896-6273 (Linking) (2010).

77. Reynolds, P., Gardner, D. & Lee, R. Tinnitus and psychological morbidity: a cross-sectional study to investigate psychological morbidity in tinnitus patients and its relationship with severity of symptoms and illness perceptions. *Clinical Otolaryngology & Allied Sciences* **29,** 628–634 (2004).

78. Rossiter, S., Stevens, C. & Walker, G. Tinnitus and its effect on working memory and attention (2006).

79. Schlee, W. *et al.* Mapping cortical hubs in tinnitus. *BMC Biol* **7,** 80. ISSN: 1741-7007 (Electronic) 1741-7007 (Linking) (2009).

80. Schneider, P. *et al.* Reduced volume of Heschl's gyrus in tinnitus. *NeuroImage* **45,** 927–39. ISSN: 1095-9572 (Electronic) 1053-8119 (Linking) (2009).

81. Scott, B., Lindberg, P., Melin, L. & Lyttkens, L. Predictors of tinnitus discomfort, adaptation and subjective loudness. *British journal of audiology* **24,** 51–62 (1990).

82. Stephens, S., Hallam, R. & Jakes, S. Tinnitus: a management model. *Clinical Otolaryngology & Allied Sciences* **11,** 227–238 (1986).

83. Stevens, C., Walker, G., Boyer, M. & Gallagher, M. Severe tinnitus and its effect on selective and divided attention: acufeno severo y sus efectos sobre la atención selectiva y dividida. *International journal of audiology* **46,** 208–216 (2007).

84. Sullivan, M., Katon, W., Russo, J., Dobie, R. & Sakai, C. Coping and marital support as correlates of tinnitus disability. *General Hospital Psychiatry* **16,** 259–266 (1994).

85. Sullivan, M. J., Bishop, S. R. & Pivik, J. The pain catastrophizing scale: development and validation. *Psychological assessment* **7,** 524 (1995).

86. Turk, D. C. & Monarch, E. S. Biopsychosocial perspective on chronic pain. (2002).

87. Tyler, R. S. & Baker, L. J. Difficulties experienced by tinnitus sufferers. *Journal of Speech and Hearing disorders* **48,** 150–154 (1983).

88. Vanneste, S. & De Ridder, D. The auditory and non-auditory brain areas involved in tinnitus. An emergent property of multiple parallel overlapping subnetworks. *Frontiers in systems neuroscience* **6,** 31 (2012).

89. Vaziri, Z. *et al.* Down-Regulation of Tinnitus Negative Valence via Concurrent HD-tDCS and PEI Technique: A Pilot Study. *Brain Sciences* **13,** 826 (2023).

90. Vlaeyen, J. W. & Linton, S. J. Fear-avoidance and its consequences in chronic musculoskeletal pain: a state of the art. *Pain* **85,** 317–332 (2000).

91. Vollmann, M., Scharloo, M., Langguth, B., Kalkouskaya, N. & Salewski, C. Illness representations as mediators of the relationship between dispositional optimism and depression in patients with chronic tinnitus: A cross-sectional study. *Psychology & Health* **29,** 81–93 (2014).

92. Wallhäußer-Franke, E. *et al.* Tinnitus: distinguishing between subjectively perceived loudness and tinnitus-related distress. *PLoS One* **7,** e34583 (2012).

93. Ware Jr, J. E. & Sherbourne, C. D. The MOS 36-item short-form health survey (SF-36): I. Conceptual framework and item selection. *Medical care,* 473–483 (1992).

94. Watanabe, M. *et al.* Attention but not awareness modulates the BOLD signal in the human V1 during binocular suppression. *Science* **334,** 829–31. ISSN: 1095-9203 (Electronic) 0036-8075 (Linking) (2011).

95. Watson, D. & Pennebaker, J. W. Health complaints, stress, and distress: exploring the central role of negative affectivity. *Psychological review* **96,** 234 (1989).

96. Weise, C., Heinecke, K. & Rief, W. Biofeedback-based behavioral treatment for chronic tinnitus: results of a randomized controlled trial. *Journal of consulting and clinical psychology* **76,** 1046 (2008).

97. Weise, C. *et al.* The role of catastrophizing in recent onset tinnitus: its nature and association with tinnitus distress and medical utilization. *International journal of audiology* **52,** 177–188 (2013).

98. Westin, V., Hayes, S. C. & Andersson, G. Is it the sound or your relationship to it? The role of acceptance in predicting tinnitus impact. *Behaviour research and therapy* **46,** 1259–1265 (2008).

99. Westin, V. Z. *et al.* Acceptance and commitment therapy versus tinnitus retraining therapy in the treatment of tinnitus: a randomised controlled trial. *Behaviour research and therapy* **49,** 737–747 (2011).

100. Wilson, P. H. & Henry, J. L. Tinnitus Cognitions Questionnaire: development and psychometric properties of a measure of dysfunctional cognitions associated with tinnitus. *The international tinnitus journal* **4,** 23–30 (1998).

101. Zenner, H. P., Pfister, M. & Birbaumer, N. Tinnitus sensitization: Sensory and psychophysiological aspects of a new pathway of acquired centralization of chronic tinnitus. *Otology & neurotology : official publication of the American Otological*

Society, American Neurotology Society and European Academy of Otology and Neurotology **27,** 1054–63 (2006).

102. Zenner, H.-P. & Zalaman, I. M. Cognitive tinnitus sensitization: behavioral and neurophysiological aspects of tinnitus centralization. *Acta oto-laryngologica* **124,** 436–439 (2004).

103. Zikopoulos, B. & Barbas, H. Circuits formultisensory integration and attentional modulation through the prefrontal cortex and the thalamic reticular nucleus in primates. *Rev Neurosci* **18,** 417–38. ISSN: 0334-1763 (Print) 0334-1763 (Linking) (2007).

104. Zikopoulos, B. & Barbas, H. Pathways for emotions and attention converge on the thalamic reticular nucleus in primates. *J Neurosci* **32,** 5338–50. ISSN: 1529-2401 (Electronic) 0270-6474 (Linking) (2012).

105. Zöger, S., Svedlund, J. & Holgers, K.-M. Psychiatric disorders in tinnitus patients without severe hearing impairment: 24 month follow-up of patients at an audiological clinic: Alteraciones psiquiátricas en pacientes con tinnitus sin hipoacusia severa: Seguimiento durante 24 meses en una clínica audiólogica. *Audiology* **40,** 133–140 (2001).

106. Zöger, S., Svedlund, J. & Holgers, K.-M. The Hospital Anxiety and Depression Scale (HAD) as a screening instrument in tinnitus evaluation. *International Journal of Audiology* **43,** 458–464 (2004).

107. Zöger, S., Svedlund, J. & Holgers, K.-M. Relationship between tinnitus severity and psychiatric disorders. *Psychosomatics* **47,** 282–288 (2006).
